# The conservation and signatures of lincRNAs in Marek’s disease of chicken

**DOI:** 10.1038/srep15184

**Published:** 2015-10-16

**Authors:** Yanghua He, Yi Ding, Fei Zhan, Huanmin Zhang, Bo Han, Gangqing Hu, Keji Zhao, Ning Yang, Ying Yu, Li Mao, Jiuzhou Song

**Affiliations:** 1 Department of Animal & Avian Sciences, University of Maryland, College Park, MD 20742, USA; 2 USDA, Agricultural Research Service, Avian Disease and Oncology Laboratory, East Lansing, MI 48823, USA; 3 Systems Biology Center, National Heart, Lung and Blood Institute, National Institutes of Health, Bethesda, MD 20892, USA; 4 Department of Animal Genetics and Breeding, National Engineering Laboratory for Animal Breeding, College of Animal Science and Technology, China Agricultural University, Beijing 100193, China; 5 Department of Oncology and Diagnostic Sciences, University of Maryland School of Dentistry, Baltimore, MD, 21201, USA

## Abstract

Long intergenic non-coding RNAs (lincRNAs) associated with a number of cancers and other diseases have been identified in mammals, but they are still formidable to be comprehensively identified and characterized. Marek’s disease (MD) is a T cell lymphoma of chickens induced by Marek’s disease virus (MDV). Here, we used a MD chicken model to develop a precise pipeline for identifying lincRNAs and to determine the roles of lincRNAs in T cell tumorigenesis. More than 1,000 lincRNA loci were identified in chicken bursa. Computational analyses demonstrated that lincRNAs are conserved among different species such as human, mouse and chicken. The putative lincRNAs were found to be associated with a wide range of biological functions including immune responses. Interestingly, we observed distinct lincRNA expression signatures in bursa between MD resistant and susceptible lines of chickens. One of the candidate lincRNAs, termed *linc-satb1*, was found to play a crucial role in MD immune response by regulating a nearby protein-coding gene *SATB1*. Thus, our results manifested that lincRNAs may exert considerable influence on MDV-induced T cell tumorigenesis and provide a rich resource for hypothesis-driven functional studies to reveal genetic mechanisms underlying susceptibility to tumorigenesis.

Mammalian conservation indicates that ~5% of the human genome^1^,^2^ is conserved due to non-coding and regulatory roles, in which more than 80% is associated with chromatin states suggestive of regulatory functions^3^,^4^,^5^. Long non-coding RNAs (lncRNAs), as non-protein coding transcripts longer than 200 nucleotides, are significantly constrained in humans^6^, suggesting primarily lineage-specific functions, while short non-coding RNAs are as strongly constrained as protein coding regions. Most (81%) lncRNA families were primate-specific. A large proportion of young lncRNAs was generated due to fast lncRNA evolution, which prevents detection of distant homologues. Interestingly, young lncRNAs possess low levels of long-term exonic sequence conservation but older lncRNAs have higher levels of conservation, implying that the later may function predominantly in lineage-specific expression^7^.

Since long intergenic non-coding RNAs (lincRNAs) are relatively easier to define, identify, quantify, and interpret compared to other lncRNA categories, recent studies have focused on characterizations of lincRNAs^8^,^9^,^10^,^11^,^12^,^13^,^14^. So far, thousands of lincRNAs have been ascertained in a variety of species^8^,^9^,^10^,^11^,^12^,^13^,^14^, sharing many common features with mRNAs, such as being capped, polyadenylated, and usually spliced^15^. Importantly, lincRNAs were involved in a wide range of biological processes including signaling, development, cell cycle, and immune response^8^,^10^. Nevertheless, a host of the lncRNAs exhibits tissue-specific expression, localization to subcellular compartments, response to stimulus, shared synteny across species, and association with disease, suggesting that they are regulated and unlikely represent transcriptional “noise”^16^,^17^,^18^,^19^,^20^.

By using next-generation sequencing (NGS), we recently have enabled unprecedented ability to detect novel transcripts by sequencing millions of short cDNA fragments^21^,^22^,^23^. Together with the advances in computational transcriptome reconstruction^24^,^25^,^26^, we are now able to comprehensively identify and characterize non-coding RNAs on a genome-wide scale. However, a fundamental but challenging task is to distinguish between protein-coding mRNAs and long non-coding transcripts^27^,^28^. To efficiently and precisely identify lincRNAs, numerous attempts have been carried out^27^,^28^. The most direct evidence for a transcript to be classified as non-coding is that no protein product is produced from the putative open reading frame (ORF); thus, one of the most frequently used criteria is the length of ORF^29^. Another strategy is to search three-frame translated peptide sequences against known protein or protein domain database (eg. Pfam^30^) using BLASTX^31^ or HMMER^32^. Moreover, short segments of sequence similar to mRNAs imply that the transcripts will be translated. Similarly, methods based on evolutionary constrains could be utilized as a threshold^28^.

It is reported that, functionally, aberrant expression of lncRNA is associated with different types of cancers and various neurological disorders^33^,^34^,^35^,^36^,^37^. LncRNAs are stable in body fluids, making them ideal candidate biomarkers for non-invasive cancer diagnosis or prognosis^33^,^38^. Recent studies showed that *HOTAIR* involved in modulating chromatin state in a number of cancers and could be a potential target for cancer diagnosis and therapy^39^,^40^,^41^. However, the efficient screening and distribution of lncRNAs are not well characterized in livestock animals, especially in chicken.

Marek’s disease (MD) is a highly contagious lymphomatous and neuropathic disease of chicken triggered by Marek’s disease virus (MDV)^42^ whose life cycle can be divided into four phases: an early cytolytic phase from 2 to 7 days post infection (dpi), a latency phase around 7–10 dpi, a late cytolytic infection starting from 18 dpi accompanying the presence of tumors and a proliferation phase after 28 dpi. However, the MD virus can evolve with emergence of new and more virulent strains resulting in loss of efficacy of current vaccines to MD. Therefore, we aim to ascertain any lincRNAs involving in Marek’s disease (MD) and to excavate ideal candidate lincRNA markers for MD diagnosis and prevention. In this study, to precisely identify lincRNAs, a series of constrains were applied to filter non-lincRNAs. Consequently, a relatively reliable and solid pipeline was developed for lincRNAs identification and potential biological function exploration. In addition, a comprehensive list of lincRNAs in chicken was identified by a rigorous pipeline based on the transcriptome sequencing data from MD resistant and susceptible chickens. By uncovering the signatures of lincRNA expression, we revealed a slice of key lincRNAs associated with MD resistance. Most importantly, we found some conserved lincRNAs with potential biological functions among different species. We believe that this study will provide valuable lincRNAs information in chicken and advance our knowledge of non-coding RNA biology, especially on MD.

## Results

### LincRNA identification in chicken

**Figure 1:**
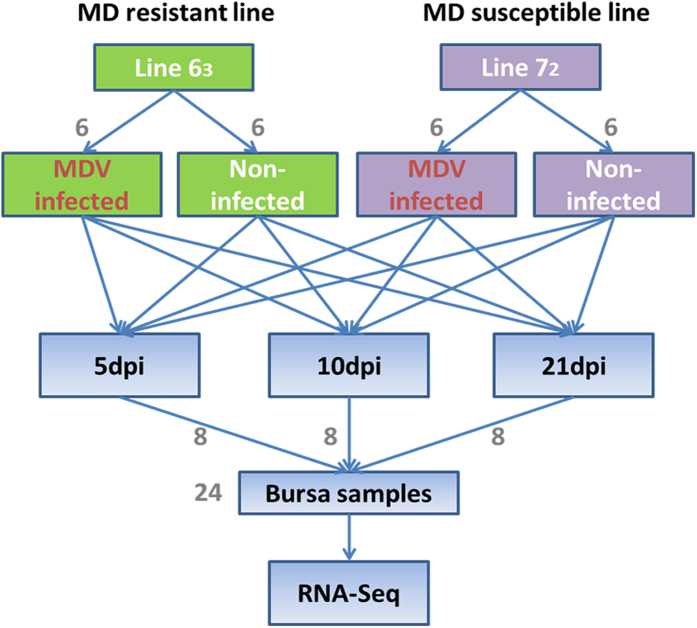
Experimental designs. Experimental design for identifying lincRNAs involved in Marek’s disease resistance using resistant chicken line 6_3_ and susceptible line 7_2_.

**Figure 2:**
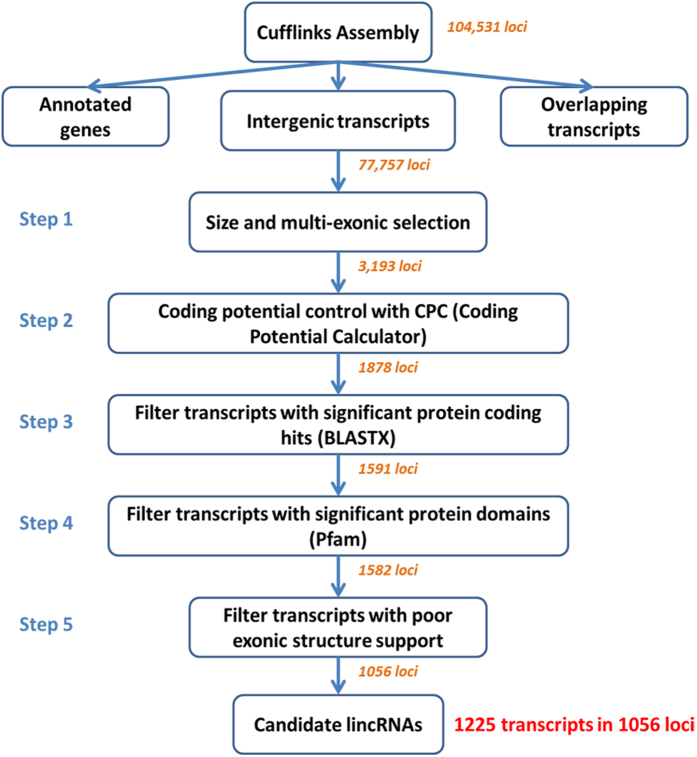
The pipeline of lincRNA identification. After transcriptome assembly with Cufflinks, intergenic transcripts were kept and then subjected to five filtering steps that remove potential and unannotated protein-coding genes and spurious transcripts. A total of 1,225 candidate lincRNA transcripts were identified in 1,056 loci. The number on an arrow indicates remaining lincRNA loci after the previous step.

To subdivide RNA molecules and identify the long non-coding transcripts, transcriptomic sequencing was conducted in 24 RNA samples extracted from chicken bursa tissue of two highly inbred lines before and after MD viral infection (Fig. 1). Approximately 600 million short reads were obtained total. By a two-iteration mapping strategy, nearly 70% of the reads were mapped to chicken genome with TopHat (Supplementary Table 1). Transcriptomes were assembled individually using Cufflinks (version 2.0.2)^24^ and merged together to build consensus transcript models. In total, 151,385 transcripts were identified in 104,531 loci across all experimental conditions. By comparing to RefSeq and Ensembl gene annotations using cuffcompare, 77,757 intergenic loci were found. Then, they were subject to five rigorous filtering steps to identify reliable candidate lincRNAs. Eventually, a total of 1,056 candidate lincRNA loci with 1,225 lincRNA transcripts were captured in our studied population (Fig. 2).

### LincRNA sequence conservation and syntenic lincRNA

**Figure 3:**
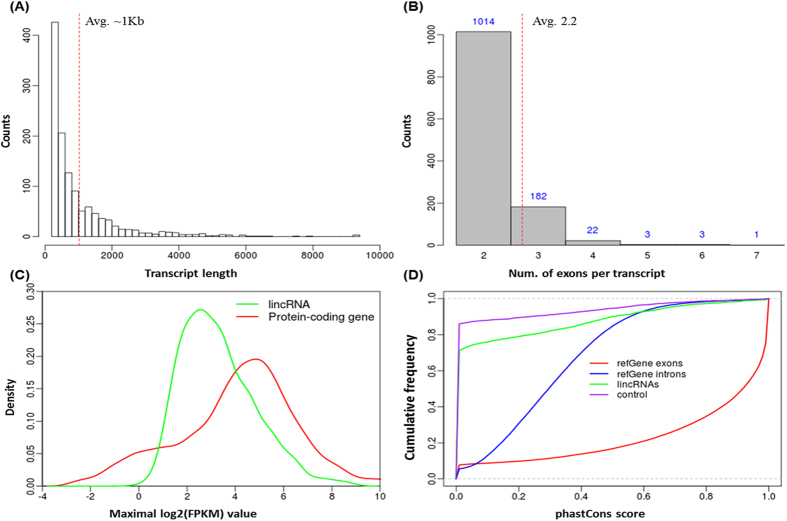
Basic properties of lincRNA. (**A**) LincRNA transcript length distribution. The average length is about 1 kb and is marked by red dot line on the Figure. (**B**) Number of exons for lincRNA transcripts. As marked by a red dot line on the Figure, on average there are 2.2 exons per transcript. (**C**) LincRNA expression levels compared to protein-coding genes. The overall expression level for lincRNAs is much lower than that of protein coding genes. (**D**) LincRNA sequence conservation measured by phastCons scores. LincRNAs show intermediate sequence conservation as compared to intergenic control regions and protein coding genes.

LincRNAs identified in this study were shorter and had fewer transcripts within each locus compared to protein-coding genes. The length of lincRNA transcripts ranged from a few hundred bases up to 9 kb, but the average length was approximately 1 kb compared to more than 3 kb for protein-coding genes (Fig. 3A). Most of the lincRNAs identified had only 2.2 exons per transcript on average (Fig. 3B) while protein-coding genes (Ensembl annotation) had averaged 21 exons, which may arise from the incomplete assembly of lincRNA transcripts with low expression levels.

To estimate lincRNA activity in our samples, the expression distribution of all lincRNAs was profiled by comparing expression levels of protein-coding genes (Fig. 3C). We found that the overall expression level of lincRNAs was approximately 8 fold lower than protein-coding genes, which was consistent with previous observations in other species^10^,^12^. Furthermore, the phastCons score^43^, which is a measure of sequence conservation based on multiple sequence alignments, was used to evaluate the sequence conservation of lincRNAs. The mean phastCons scores were calculated for lincRNAs in the transcript regions, exons and introns of RefSeq genes, and control regions for lincRNAs, respectively (Fig. 3D). The control set for lincRNAs was generated based on 1,225 transcripts. As expected, the control regions manifested the lowest conservation while RefSeq exonic and intronic regions possessed stronger and moderate sequence conservation, respectively, as compared to lincRNAs. Overall, lincRNAs identified in our study enjoy fewer exons, shorter transcript length, significantly lower expression levels, and lower sequence conservation compared to protein-coding genes, which were consistent with studies in other species^10^,^11^,^12^,^13^.

**Figure 4:**
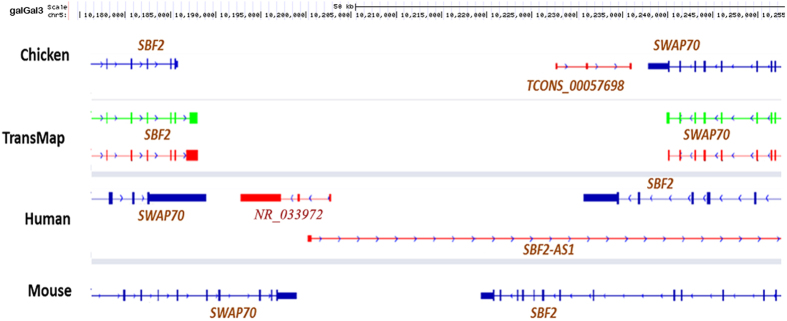
A representative syntenic lincRNA. Transcript TCONS_00057698 is a lincRNA identified in chicken and locates in the upstream of *SWAP70* with opposite transcriptional direction. In the human genome, an annotated non-coding RNA, NR_033972, is positional equivalent with TCONS_00057698 in chicken, and both non-coding RNAs have three exons. But, in mouse genome, no positionally equivalent non-coding RNA was identified.

**Figure 5:**
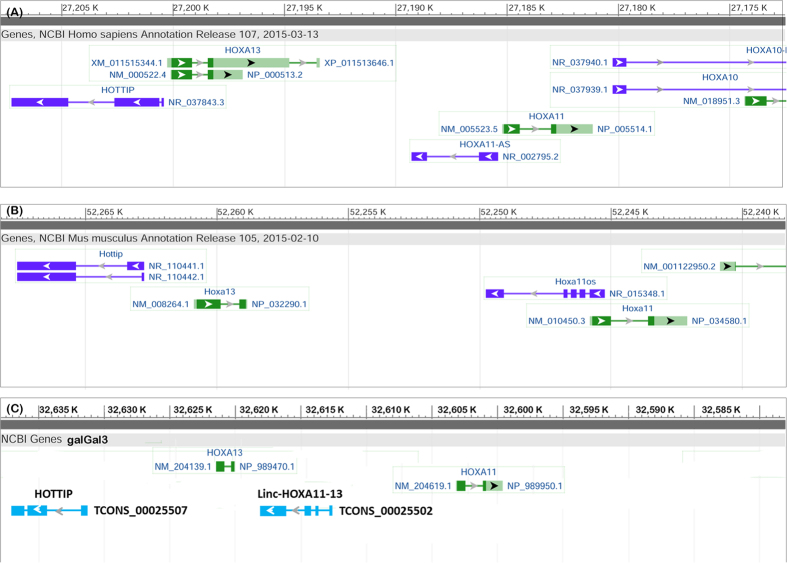
Two lincRNA orthologs located in the HOXA cluster on chicken genome. (**A**) HOTTIP locates at upstream of HOXA13 gene and HOXA11-AS between HOXA13 and HOXA11 in human genome. (**B**) Two lincRNAs, HOTTIP, and HOXA11OS in mouse genome. (**C**) In chicken genome, one positionally equivalent lincRNA locus was identified as the HOTTIP ortholog and another lincRNA ortholog linc-HOXA11-13 locating between HOXA11 and HOXA13 was identified nearby.

**Figure 6:**
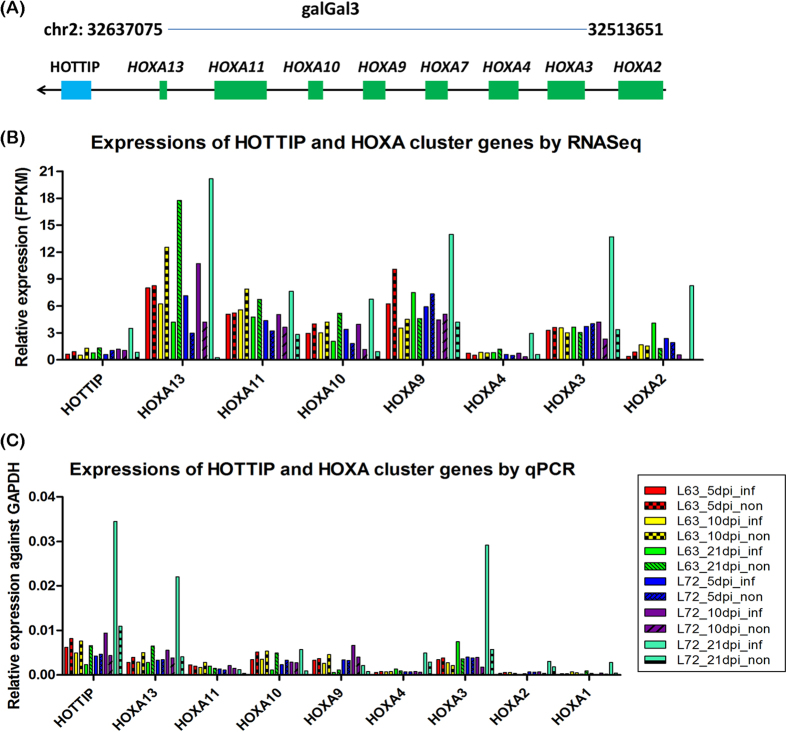
The expressions of *HOTTIP* homolog and *HOXA* cluster genes across all chickens with different conditions for two chicken lines. (**A**) The schematic diagram of *HOTTIP* and *HOXA* cluster genes on chicken genome (galGal3). (**B**) Expressions of *HOTTIP* and *HOXA* cluster genes were determined by RNA sequencing and the result showed that *HOTTIP* homolog in chicken was positively correlated with *HOXA* genes in the cluster for the same individual. Besides, with the increased distance to *HOTTIP* (from *HOXA13* to *HOXA2*), expressions of *HOXA* genes would gradually decrease.

To investigate the evolutionary conservation of the lincRNAs, we compared the identified chicken lincRNAs to human and mouse. In addition to sequence similarity, potential lincRNA orthologs were also identified via searching positional equivalent non-coding RNAs in the human and mouse genome (Fig. 4). For 759 lincRNA loci located on autosomes, TransMap refGene of chicken was used to determine orthologous genes in human and mouse for protein-coding neighbors of lincRNAs. We found that around 75% (594 and 560 for human and mouse, respectively) of neighboring protein-coding gene pairs can be found having human or mouse orthologs, which locate adjacently to each other. Furthermore, the putative syntenic lincRNA orthologs of chicken were identified by searching non-coding RNAs located between orthologous gene pairs. Approximately 312 lincRNAs of chicken were observed having positional equivalent non-coding RNAs on the human or mouse genomes, in which, most of these potential syntenic lincRNA orthologs were annotated as miscRNAs and 25 were annotated as microRNAs and ncRNA. However, many lincRNAs were annotated well in the human genome, in which, two lincRNA orthologs located in the *HOXA* cluster emerged from these positional equivalent non-coding RNAs (Fig. 5). *HOTTIP* that is located at the 5′ distal tip of the *HOXA* locus is one of few well-characterized mammalian lincRNAs (Fig. 5A)^44^. In chicken genome, one positionally equivalent lincRNA locus was identified as the *HOTTIP* ortholog and another lincRNA ortholog *HOXA11-AS*, locating between *HOXA11* and *HOXA13* was identified nearby (Fig. 5C). Besides, these two lincRNAs were also found as positionally equivalent orthologs on the mouse genome (Fig. 5B) indicating that they are older lincRNAs and highly conserved during the independent evolution. To reveal the relationship between *HOTTIP* and *HOXA* cluster in chicken, the expression correlation of *HOTTIP* homolog in chicken was inspected with *HOXA* cluster; we found that *HOTTIP* homolog in chicken was positively correlated with *HOXA* genes in the cluster (Fig. 6), which was consistent with the *HOTTIP* as a 5′ *HOXA* gene activator^44^. Interestingly, with the increased distance to the lincRNA (from *HOXA13* to *HOXA1*), the correlation coefficient decreased as well, which may be explained by the *cis* regulatory nature of *HOTTIP* lincRNA. Additionally, we identified 49 (~6%) chicken lincRNAs mapped to human or mouse protein-coding regions, which these lincRNAs were probably unannotated protein-coding genes or derived from ancestral protein-coding genes.

### LincRNA functions in chicken

**Figure 7:**
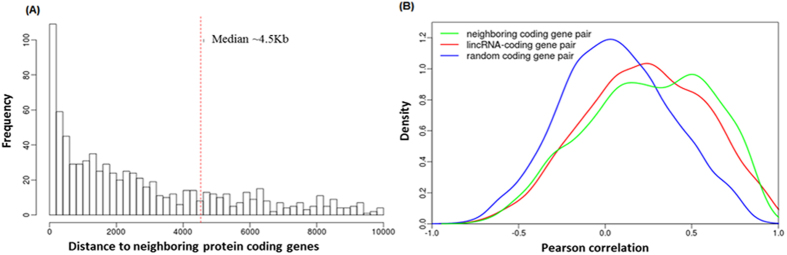
LincRNAs and neighboring protein-coding genes. (**A**) The distribution for the distance from lincRNAs to their neighboring protein-coding genes. The median distance is about 4.5 kb as marked by a red dot line. (**B**) The comparison of expression correlation. Pearson correlation coefficients were calculated for (a) neighboring protein-protein coding genes pair, (b) lincRNA loci and their nearest protein-coding genes pair (c) randomly protein-coding genes pair. A density plot was generated based on correlation coefficients for all three groups.

To explore lincRNA functions in chicken, the relationship between lincRNAs and neighboring protein-coding genes was firstly uncovered via the distance and expressions between them. For each lincRNA locus, we identified its adjacent upstream and downstream protein-coding genes. The distances from lincRNAs to their neighboring genes ranged from a few bases up to 5 Mb with a median distance of about 4.5 Kb, and approximately 65% of the protein-coding neighbors were within 10 Kb (Fig. 7A). To test whether lincRNAs are co-expressed with protein-coding neighbors, Pearson correlations of expression levels between lincRNAs and neighboring protein-coding genes were calculated. We observed stronger expression correlations between lincRNAs and their neighboring protein-coding genes than for randomly selected protein-coding gene pairs (Fig. 7B; Kolmogorov-Smirnov test). However, there was no significant difference in correlation compared to neighboring protein-protein coding genes pairs. This result is essentially in agreement with previous studies in human, mouse and zebrafish^10^,^12^,^45^.

**Figure 8:**
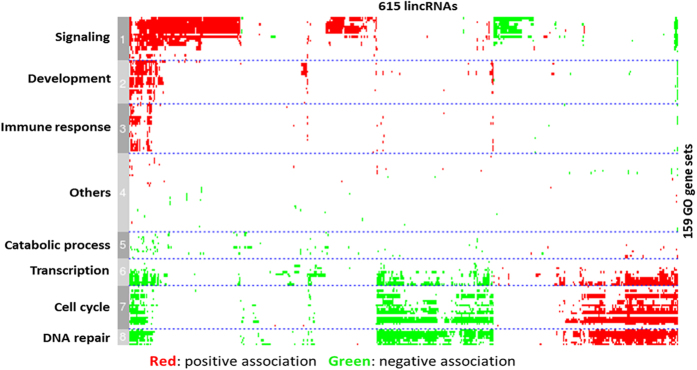
Computational lincRNA function annotation. Expression-based association matrix of 615 lincRNA loci (column) and 159 functional genes sets (row). Red represents positive association, green represents negative association, and white represents no significant association. Based on GO gene sets, the matrix was classified into 8 clusters and GO function for each cluster is labeled on the left.

To test whether lincRNAs were preferentially located in the vicinity of protein-coding genes with specific functions, Gene Ontology (GO) enrichment was analyzed by DAVID for lincRNA neighboring genes within 10 kb. Our results indicated that those neighboring genes were significantly enriched (p < 0.005) in pathways, such as cell death, vasculature development, regulation of transcription, gland development, regulation of RNA metabolic process, and lymphocyte activation (Supplementary Table 2). Based on the expression correlation of lincRNA with protein-coding genes, our results demonstrated that lincRNAs may involve in biological processes including regulation of transcription, signaling and regulation of development^8^,^10^,^12^,^46^. To computationally assign functions to the identified lincRNAs, we constructed a correlation matrix by calculating Pearson correlations of expressions between lincRNA loci and protein-coding genes across twelve groups (2 chicken lines, 2 infection treatments, and 3 time-points). An association matrix was built between 615 lincRNA loci and 159 significantly enriched functional gene sets; 8 distinct functional groups were generated in which gene sets sharing similar annotations were clustered to one group based on GO functional terms. Strong correlations were found between lincRNA loci and certain groups of protein-coding genes (Fig. 8). As shown in Fig. 8, we found several sets of lincRNAs associated with functional groups such as signaling, cell cycle, development, transcription, DNA repair and immune response. Of the annotated lincRNAs (615 in total) more than 30% were manifested association with signaling. For the rest of lincRNAs, most of them were associated with cell cycle, DNA repair and transcription. Almost the same set of lincRNAs was involved in all three functional groups, which may be explained by the fact that these three functions are highly related. Although most lincRNAs were only associated with specific functions, a small portion of lincRNAs (~45) demonstrated a wide spectrum of functionality and was associated with multiple distinct functional groups.

### LincRNA expression signatures in MD resistant and susceptible chickens

**Figure 9:**
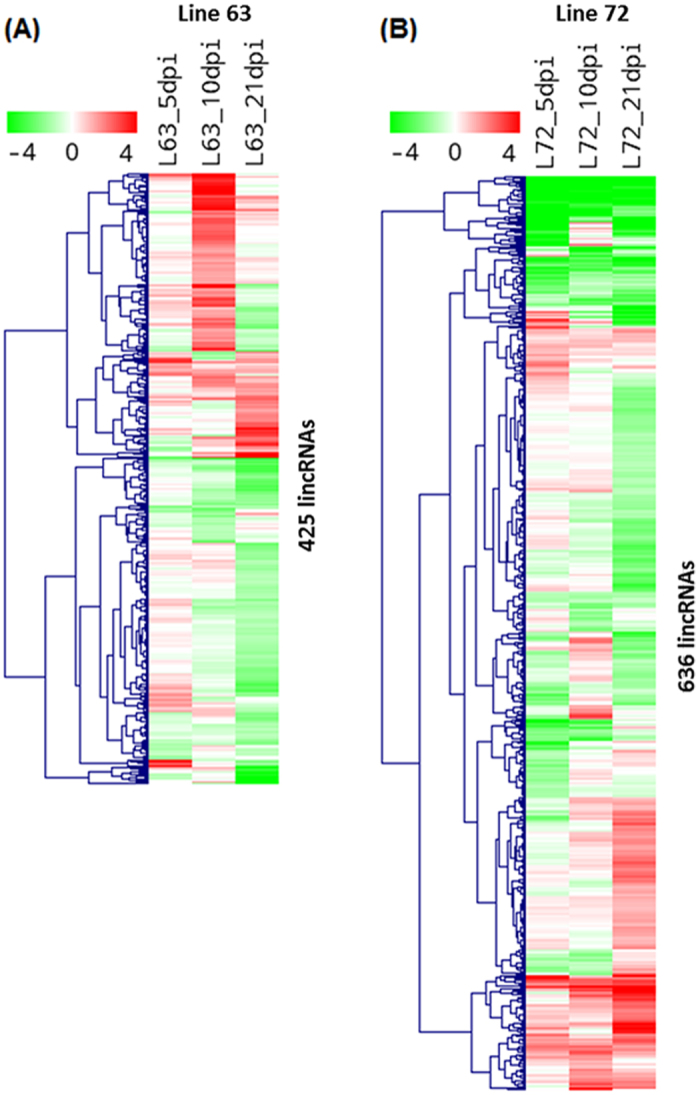
LincRNA expression signatures. Expression profiles of differential expressed lincRNAs between infected and non-infected chickens at three time-points of each line. LincRNAs were indicated as rows and different experimental conditions were indicated as columns. Each value represents log2 ratio of a lincRNA expression level in infected chicken compared to non-infected chicken. The matrix was clustered by lincRNAs using hieratical clustering with complete linkage. Red indicates up-regulation and green represents down-regulation. (**A**) Expression profile for 425 differentially expressed lincRNAs in line 6_3_. (**B**) Expression profile for 636 differentially expressed lincRNAs in line 7_2_.

**Figure 10:**
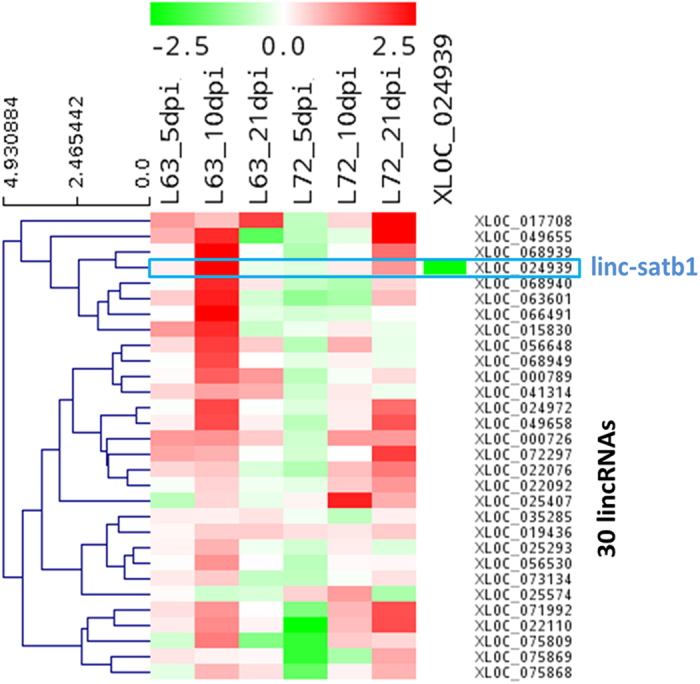
Expression signatures of lincRNAs related to immune response. Expression profile for 30 lincRNAs that were annotated involving immune response through co-expression based functional annotation. LincRNAs were represented as rows and different experimental conditions were represented as columns. Each value represents log2 ratio of a lincRNA expression level in infected chicken compared to non-infected chicken for specific chicken line and time point. Red indicates up-regulation and green represents down-regulation. The matrix was clustered by lincRNAs using hieratical clustering with complete linkage.

To systematically investigate the expression changes of lincRNAs induced by MDV infection and reveal whether lincRNAs are associated with Marek’s disease, we identified differentially expressed lincRNAs between infected and non-infected chickens at three time-points for each line. A total of 425 and 636 differential lincRNA loci were identified (FPKM of both >1 and fold change ≥2) at least one time point for line 6_3_ and line 7_2_, respectively (Fig. 9). Besides, differential protein-coding genes were also identified in these two chicken lines, and a total of 387 and 2383 differentially expressed genes (FDR ≤ 0.05 and fold change ≥2) were found between infected and non-infected chickens in line 6_3_ and line 7_2_, respectively. For line 6_3_, 92% of differentially expressed genes were up-regulated after MDV infection, which involved in Cytokine-cytokine receptor interaction and Cell adhesion molecules (CAMs) pathways (p-value < 0.01) including CD family genes, chemokine receptor and interleukin receptor genes as well as tumor necrosis factor receptor superfamily genes (Supplementary Fig. 1-2). The notable point is that these differentially expressed genes associated with immune response were expressed highly in infected chickens of line 6_3_ at only 10 dpi while they were highly expressed in infected chickens of line 7_2_ at 21 dpi (Supplementary Table 3). Based on functional correlation analysis between lincRNA loci and protein-coding genes (Fig. 8), 30 lincRNAs associated with immune response were extracted and their expression profiles were plotted (Fig. 10) across different time-points and chicken lines. The nearest genes of these lincRNAs were showed in Supplementary Table 4 and most of these genes are associated with immune response. Interestingly, distinct expression patterns for these 30 lincRNAs were observed between resistant line 6_3_ and susceptible line 7_2_. For resistant line 6_3_, these lincRNAs were mostly up-regulated in infected chickens at 5 dpi and highly expressed at 10 dpi, in the contrary, most lincRNAs were down-regulated after infection and up-regulation was mostly seen at 21 dpi for susceptible line 7_2_. But most importantly, there were similar expression patterns for these 30 lincRNAs and differentially expressed genes. MDV goes through early cytolysis at 5 dpi, a latency phase at 10 dpi and a late cytolytic infection at 21 dpi. Therefore, for resistant line 6_3_, lincRNAs related to immune responses started to act in the early or latency phase of MDV instead of the late phase of 21 dpi for susceptible line 7_2_, which suggested that these immune-related lincRNAs are associated with the resistance of MDV and could act on protein-coding genes involving in immune responses by *cis* or *trans* in tumorigenesis of Marek’s disease.

### Key lincRNAs involved in Marek’s disease resistance

**Figure 11:**
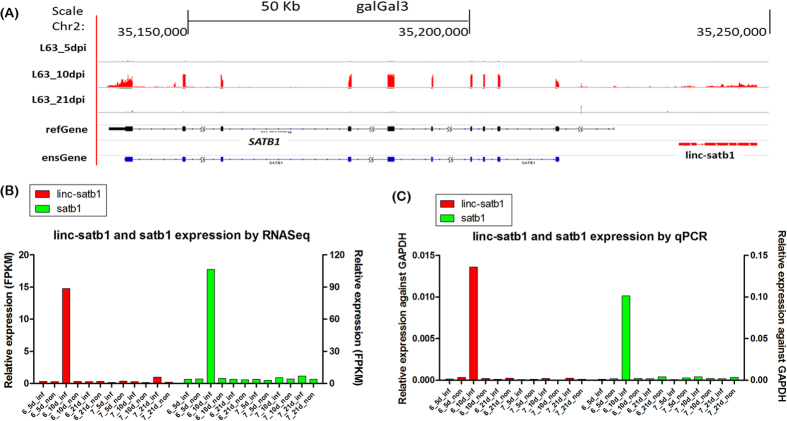
LincRNA *linc-satb1* probably associated with immune response to Marek’s disease. (**A**) Relative genomic location was shown for *linc-satb1* (red) and *SATB1* gene (black for refGene annotation, and blue for Ensembl annotation). Expression levels in line 6_3_ chickens at 5, 10, and 21 dpi were shown in the above three panels. (**B**) Expression levels for *linc-satb1* (left-y-axis) and protein-coding gene *SATB1* (right-y-axis) were shown across all groups based on RNA-Seq data. (**C**) Expression validation for lincRNA *linc-satb1* (left-y-axis) and for protein-coding gene *SATB1* (right-y-axis) in different treatment groups by qPCR assay.

The exciting thing is a lincRNA (*linc-satb1*) that may associate with Marek’s disease resistance was identified in the upstream region of the *SATB1* gene (Fig. 11A). In order to confirm that linc-satb1 is truly non-coding, we checked Kozak consensus sequence (gcc)gccRccAUGG in linc-satb1 sequence and the result showed that there was no efficient and reliable Kozak sequences for initiation of translation^47^ (Supplementary Table 5). Based on expression correlation with other protein-coding genes, *linc-satb1* was found to positively relate to defense response, inflammatory response, lymphocyte activation and response to external stimulus; but to negatively associate with cell cycle-related functions such as cell cycle process and DNA replication. Moreover, *linc-satb1* was only highly expressed in infected birds of MD-resistant line 6_3_ at 10 dpi (Fig. 10 and Fig. 11B) corresponding to the latent phase of MDV infection. All these observations implied that *linc-satb1* was probably associated with immune response to MDV infection.

A strong positive correlation (Pearson correlation coefficient ~0.95) was observed on expression levels between *linc-satb1* and its nearby protein-coding gene *SATB1* (Fig. 11C). *SATB1* is a genome organizer that regulates chromatin structure and a transcription factor that controls a large number of genes involved in T cell development and activation^48^,^49^. So far, most of research reports that *SATB1* is associated with malignant neoplasms, tumor progression and carcinogenesis diseases (Supplementary Fig. 3), which further implies that *SATB1* might relate to Marek’s disease. Besides, Gene Set Enrichment Analysis of target genes of SATB1^50^ indicated that no matter for up-regulated genes or down-regulated genes after knockdown of *SATB1*, nearly 50% target genes were significant enriched between infected and non-infected chickens, but more up-regulated target genes (47.7%) were enriched at 10 dpi compared to down-regulated target genes (32.6%), which suggested that *linc-satb1* could be a mediator to act on *SATB1* gene for up-regulating target genes at 10 dpi in line 6_3_ during progression of Marek’s disease (Supplementary Data, Supplementary Table 6). Notably, we found that *LEF1* and *TCF7* as T lymphocyte associated activators and up-regulated target genes of *SATB1* were highly induced in infected birds of line 6_3_ at 10 dpi. More importantly, cytotoxic T cell co-receptor *CD8A* and *CD8B* were only highly expressed in line 6_3_ birds at 10 dpi, which was in agreement with the expression pattern of *SATB1* and *linc-satb1* (Supplementary Fig. 4). These two glycoproteins are of great importance in cell-mediated immunity, including cytotoxic T cells recognition to infected cells by binding to MHC class *I* molecules that display virus antigens. CD8 + T cells then induced apoptosis of infected cells and prevented tumorigenesis. Thus, these genes related to T lymphocytes would be the key “candidates” to study the resistance of Marek’s disease.

**Figure 12:**
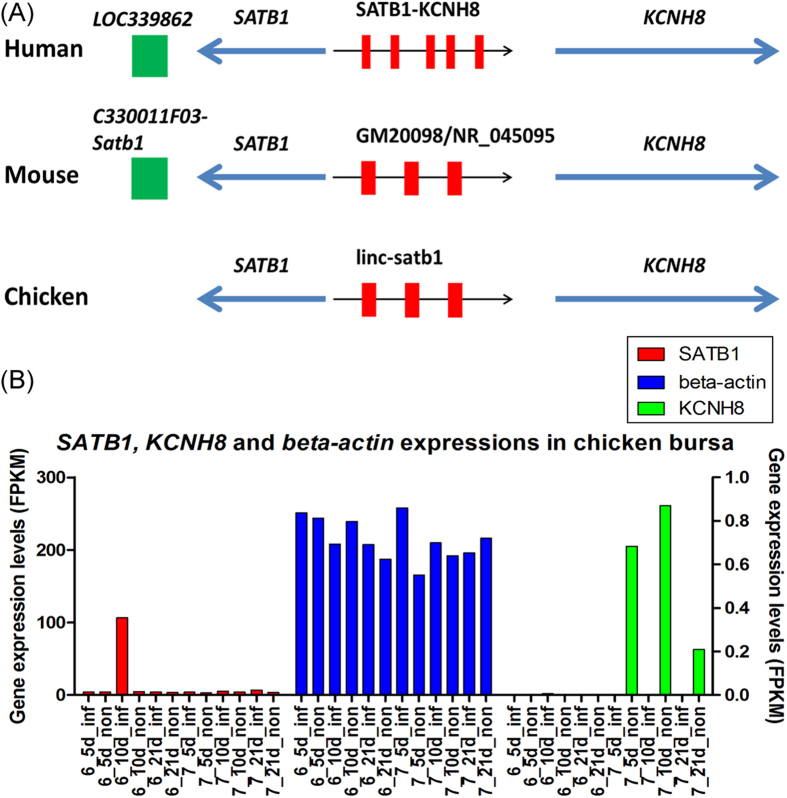
*linc-satb1* and neighboring protein-coding gene *SATB1* and *KCNH8*. (**A**) *linc-satb1* in chicken and its orthologs in human and mouse. SATB1 locates upstream of *linc-satb1* and its orthologs or *KCNH8* locates downstream of *linc-satb1* across chicken, human and mouse. Red boxes represent transcripts of lincRNAs. The arrows indicate transcriptional directions. On genome of human and mouse, there is one enhancer on downstream of *SATB1* gene (green boxes). (**B**) *SATB1* and, *beta-actin* expressions (for left-y-axis) as well as *KCNH8* (for right-y-axis) in chicken bursa across 12 individuals.

Moreover, a potential homolog of *linc-satb1*, termed as GM20098/NR_045095, was found in mouse genome. GM20098 was annotated as a validated miscRNA and has the same synteny in chicken with *linc-satb1*. Besides, *linc-satb1* and GM20098 have three exons and comparable transcript length. Also, GM20098 has an identified negative enhancer Gm20098-Kcnh8 in mouse genome and corresponding SATB1-KCNH8 in human genome, but in downstream of *satb1* gene, there is one positive enhancer LOC339862 identified in human transgenic embryos assay and orthologous enhancer C330011F03-Satb1 in mouse (Fig. 12A and Supplementary Table 7-8), which implies that this lincRNA (C330011F03-Satb1) as a positive enhancer might coordinate oppositely expressions of *SATB1* and *KCNH8* with negative non-coding RNAs (Gm20098-Kcnh8) in concert. Our data indicated that *SATB1* gene was significantly expressed in only infected chickens at 10 dpi of line 6_3_ but *KCNH8* gene was expressed in non-infected chickens of line 7_2_, although their expression levels were not higher than those of *beta-actin* (Fig. 12B). These observable results revealed that *SATB1* could promote tumor emergence and progression while *KCNH8* could regulate physiological response to MD virus^51^. At the same time, *linc-satb1* as a mediator would enhance *SATB1* functions and balance interactions of related genes. Therefore, *linc-satb1* may play a crucial role in the immune response to Marek’s disease and could be a key candidate marker for the study of Marek’s disease.

### LincRNA validations

**Figure 13:**
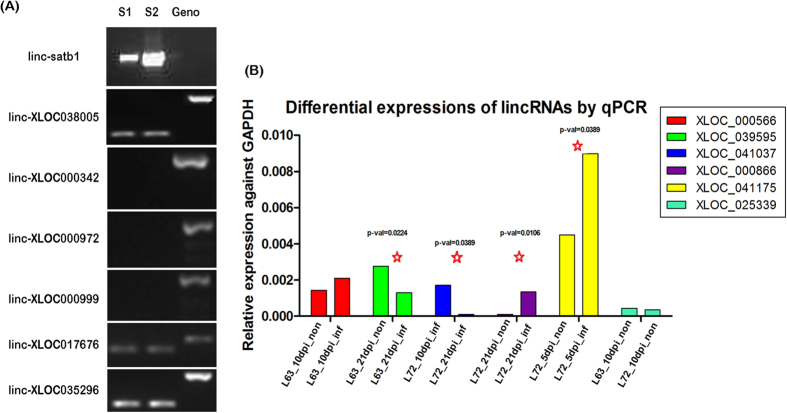
The validation of exonic structure and differential expressions of candidate lincRNAs. (**A**) The validation of lincRNA structure using ordinary PCR. Lane S1 and S2: amplified fragments for target lincRNAs with dscDNA as the template and corresponding lincRNAs were indicated on the gel; Lane Geno: amplified fragments for specific lincRNAs with chicken genomic DNA as the template. (**B**) The validation of differentially expressed lincRNAs using qPCR assay. Different comparisons were labeled on the graph. LincRNA loci with more than 2-fold changes are considered differentially expressed and are marked by a red star. Also, the differentially expressed lincRNAs were labelled with p-value.

To verify the identified lincRNAs, we first tested transcript structures of 20 lincRNA transcripts in 14 loci by PCR reaction with dscDNA as template from 24 individual samples. The results indicated that five lincRNA structures including *linc-satb1* were confirmed against chicken genomic DNA as control sample that was capable of amplifying one or more fragments in the chicken genome (Fig. 13A). In order to confirm differentially expressed lincRNAs among different treatment groups, quantitative PCR (qPCR) of 14 lincRNAs in various treatment conditions were conducted and 80% lincRNAs manifested significant differential expression (Fig. 13B and Supplementary Fig. 5).

Based on previous information, qPCR for lincRNA *linc-satb1* (XLOC_024939) and *SATB1* gene was performed to confirm co-expression of *linc-satb1* and *SATB1* (Fig. 11B,C). The results were consistent with RNA-Seq in which *linc-satb1* and *SATB1* displayed significantly high expression in infected chickens of line 6_3_ at 10 dpi in contrast to other treatment groups.

## Discussion

In this study, transcriptomic sequencing was used to interrogate the whole transcriptome of chicken bursa over three time-points corresponding to critical phases of MDV infection. By using *ab-initio* transcriptome assembly followed by stringent lincRNA identification criteria, more than 1,000 candidate lincRNA loci were ascertained in MD chickens. We found that the identified lincRNAs shared similar properties to those reported in mammalian genomes, including significant lower expression, shorter transcript length, fewer exons, and lower sequence conservation when compared to known protein-coding genes. In accordance with previous reports^10^,^12^,^13^, higher correlation for expression profiles was not detected between lincRNAs and their nearby protein-coding genes than between adjacent protein-protein coding genes. In addition, GO term enrichment analysis of lincRNA neighboring genes indicates that lincRNAs were preferentially located in the vicinity of protein-coding genes that are related to apoptosis, cell death, and transcription regulation in our study.

We found that the sequence conservation of lincRNAs was significant lower than that of protein-coding regions. Despite the relative low sequence conservation, shared synteny was detected for a number of chicken lincRNAs in human and mouse, including well-characterized lincRNAs such as *HOTTIP*. It was reported that <6% of zebrafish lincRNAs owned detectable sequence conservation with human or mouse lincRNAs^13^ and only ~12% of human and mouse lincRNAs appeared to be conserved in the other species^12^,^52^. These results suggested that unlike protein-coding mRNA that bears high selection pressure to preserve synonymous amino acid sequences, lncRNAs are probably subject to constrains by structure or sequence-specific interactions to preserve only short sequence regions^53^. For example, long non-coding RNAs can function as guides (eg. *Xist*) and scaffolds (eg. *HOTAIR*) of histone modification complexes to form the RNA-protein complexes that influence the regulation of genes expressions^40^,^54^. Accordingly, these conserved RNA domains may guide specific binding of proteins with DNA or RNA sequence.

Interestingly, lincRNAs may exert their regulatory functions by aiding the establishment of open chromatin domains to promote the accessibility or protein-coding genes to RNA polymerases^55^,^56^, and may also be post-transcriptionally processed to yield many small RNAs. For example, H19 hosts miR-675 in its first exon^57^,^58^. Ten highly conserved snoRNAs were derived from lncRNA Gas5^59^. To check whether small RNAs were derived from lincRNAs in our case, miRNA and snoRNA domains were identified in our lincRNA catalogs. By searching against Rfam database^60^, we found that significant miRNA and snoRNA domains were derived from seven and five lincRNAs, respectively, which were consistent with the annotations of syntenic lincRNAs in chicken mentioned above, indicating that these lincRNAs may be precursors for small regulatory RNAs (Supplementary Table 9). Consequently, some lincRNA transcripts would be processed to yield quite a bit of conserved small RNAs with likely unique biological functions. Besides, based on the co-expression analysis between lincRNA and protein-coding genes, lincRNAs in MD were mainly involved in signaling, cell cycle, development, transcription regulation, DNA repair, and immune response. Previous research from functional genomics has demonstrated that Marek’s disease increases DNA damage and oxidative stress in chickens^61^. In this study, a large number of lincRNAs were differentially expressed both between infected and non-infected chickens and between different chicken lines. Functional enrichment analysis of lincRNAs revealed distinct expression patterns between resistant line 6_3_ and susceptible line 7_2_, implying immune-related lincRNAs could play critical roles in MD. Especially, a lincRNA termed *linc-satb1* that may associate with MD resistance was identified as highly expressed in infected birds of line 6_3_ at latency stage. Functionally, it was positively associated with GO terms such as defense response, inflammatory response, lymphocyte activation, and response to external stimulus, implying its role in immune response to MDV infection. *Linc-satb1* may exert its function by activating the expression of *SATB1* gene because strong expression correlation was observed between *linc-satb1* and *SATB1*. Because *SATB1* organizes the MHC class-I locus into distinct chromatin loops, we think that *SATB1* with higher expression may induce a large number of immune genes up-regulated, thus, cell-mediated immunity may be activated to destroy MDV infected cells. Moreover, like many well-characterized lncRNAs, *linc-satb1* may activate *SATB1* by recruiting chromatin-modifying complexes in *cis*. Nevertheless, other mechanisms of *linc-satb1* regulation also could be feasible. For instance, *linc-satb1* could act in *trans* to regulate target genes of *SATB1* located in distal regions. Overall, our analysis indicates that *linc-satb1* mediated *SATB1* gene activation may partially explain the difference in immune response to MDV between these two chicken lines. To deeply investigate the function of *linc-satb1*, further studies are indispensable to discern the acting pathways of *linc-satb1* in the resistance of Marek’s disease via specific experimental assays, such as overexpression or RNA immunoprecipitation (RIP).

## Materials and Methods

### Chicken lines and experimental design

Line 6_3_ and line 7_2_ from USDA-ARS Avian Disease and Oncology Laboratory (ADOL, East Lansing, Michigan, USA), which are MD-resistant and MD-susceptible, respectively, were obtained. For each line, the chickens were divided into two groups with six chickens infected by MDV and six uninfected controls. A very virulent plus (vv+) strain of MDV (648A passage 40) was injected intra-abdominally on the fifth day after hatching with a dosage of 500 plaque-forming units (PFU). Bursa of Fabricius was collected at 5, 10, and 21 dpi and stored in RNAlater (Qiagen, USA) immediately, and then stored at −80 °C until RNA extraction (Fig. 1). Proper procedures in animal challenge trials were followed according to ACUC guidelines established and approved by the ADOL ACUC (April 2005) and the Guide for the care and use of Laboratory Animals by Institute for Laboratory Animal Research (2011).

### Library preparation and transcriptome sequencing

Total RNA was extracted using the standard TRIzol (Invitrogen) protocol and mRNA isolation was performed by Oligotex mRNA Mini Kit (QIAGEN). Then mRNA was used to synthesize the first and the second strand cDNA by using SuperScriptTM III Reverse Transcriptase (Invitrogen, Carlsbad, CA, USA) and oligo (dT) 12–18 primers (Invitrogen, Carlsbad, CA, USA). After purification, the double-strand cDNA (dscDNA) was fragmented into ~300 bp. Then the library for sequencing on the Illumina HiSeq 2000 Analyzer was performed as follow. End repair of the fragmented dscDNA was performed and then 3′ poly-A was added to the end-repaired dscDNA by DNA polymerase I, Large (Klenow) Fragment. A pair of Solexa adaptors was ligated to the repaired ends by T4 ligase and then 200–400 bp of fragments were selected on the Invitrogen® 2% E-Gel. Specific dscDNA fragments were amplied by PCR and the libraries were then quantified and pooled. Finally, cluster generation and sequencing analysis were performed on the Illumina Hiseq 2000 following manufacturer protocol.

### Public data sources

Chicken genome assembly galGal3 (WUGSC 2.1, May 2006), refGene annotation (galGal3, Feb 2013) and phastCons7way table^43^ were downloaded from UCSC Genome Browser (http://genome.ucsc.edu/index.html). Chicken ensGene (WASHUC2.70) annotation was downloaded from Ensembl Genome Browser (http://useast.ensembl.org/index.html). TransMap RefGene for human and mouse were obtained from UCSC Genome Browser. GenBank annotations for human and mouse from NCBI (http://www.ncbi.nlm.nih.gov/) were used for syntenic ortholog identification.

### Mapping and assembly of transcriptomic data

Transcriptomic sequencing reads for each sample were mapped to chicken genome individually using the spliced read aligner TopHat (version 2.0.6)^62^. To maximize the use of exon junction information derived across all samples, a two iteration mapping strategy was used. In the first iteration, sequencing reads of 24 samples were mapped to the chicken genome (galGal3) using TopHat with ‘—max-intron-length’ set to 200 kb and other parameters set to default. For the second iteration of mapping, all reads were re-mapped to the genome with exon junctions identified in the first run.

Mapped transcripts were assembled individually with Cufflinks, which was run with ‘—GTF-guide’. Ensembl gene annotation was supplied to guide RABT (Reference Annotation Based Transcript) assembly. Besides, options ‘-frag-bias-correct’ and ‘-multi-read-correct’ were turned on to improve transcript abundance estimation. Transcripts from all samples were then merged together with cuffmerge to build a consensus set of transcripts across samples.

### The pipeline of lincRNA identification

To identify long intergenic non-coding RNAs (lincRNAs), transcripts were compared to genome annotations (ensGene and refGene) with Cuffcompare to exclude those that overlap with protein-coding genes, pseudogenes, and ncRNAs other than intergenic transcripts. Remaining transcripts located in the intergenic regions were then adapted to the following five filters to identify candidate lincRNAs. (1) The selection of size and multi-exons: we removed suspicious single exon transcripts that may originate from nonspecific transcription initiated by Pol II or DNA contaminations in the cDNA library. Besides, short transcripts less than 200 bp were excluded. (2) The control of protein coding potency: protein-coding potential score was calculated for each transcript on both forward and reverse strand by using coding potential calculator (CPC)^63^. The CPC distinguishes protein-coding transcripts from non-coding RNAs based on the homology and open reading frame features of the input transcripts and a score between −1 and 1 will be calculated for each transcript. A transcript is classified as non-coding if potential scores for both forward and reverse strands are less than zero. If any of the transcripts within a candidate lincRNA locus is classified as protein-coding, this locus will be excluded. (3) The control of significant protein coding hits: all putative lincRNAs were searched against a non-redundant protein database using BLASTX^31^ to filter out transcripts derived from unannotated protein-coding genes. Hits that are longer than 30 bp and e-value less than 0.001 are considered significant protein-coding hits. A lincRNA locus will be kept if none of the transcripts in the loci has any significant protein-coding hits. (4) The control of significant protein domains: all transcripts were translated into amino acid sequences through all three reading frames to remove transcripts that contain known protein domains. HMMER^32^ was used to identify any known protein domain by searching against the Pfam database (Pfam 27.0)^30^ and transcripts with significant Pfam hits were excluded. (5) The control of poor exonic structure support: the validity of the identified lincRNA transcripts relies on the quality of spliced reads. Because quite a few candidate lincRNAs spanning their exon junctions have poor sequencing quality or mapping quality, the average mapping quality score of mapped spliced reads and the number of spliced reads with highest quality (50) were checked for each putative lincRNA transcript. Those candidate lincRNAs without any mapped spliced read of quality 50 or with average quality score less than 10 were discarded. Finally, candidate lincRNAs were reliably identified by these series of filtering steps.

### Gene expression estimation and normalization

The expression levels of all protein-coding genes and lincRNAs were estimated using Cufflinks^24^ by its expression abundance estimation mode with upper quantile normalization, in which refGene and ensGene annotations were combined together to get more comprehensive coverage of annotated genes. The expression levels were represented with FPKM (Fragments Per Kilobase of transcript per Million mapped reads)^24^ and log2 transformation was taken for normalization. To get the expression patterns of lincRNAs, log2 fold change between infected and non-infected chicken was calculated at different time-points for each chicken line. Those lincRNAs with greater than 1 FPKM of expression values across all three time-points and with fold changes greater than or equal to 2 in at least one time point were selected for clustering of expression profiles based on hierarchal clustering with complete linkage and visualized using heatmaps.

### Sequence conservation of lincRNA

To assess the sequence conservation of lincRNAs, phastCons7way table from UCSC was used. PhastCons7way table contains sequence evolutionary conservation information generated from Multiz alignments of 7 vertebrates. PhastCons scores^43^ were calculated using a phylogenetic hidden Markov model, and the score is higher the sequence conservation is relative highly due to a lower selection pressure. For each lincRNA, we calculated the mean phastCons score in the transcript region. Similarly, we also calculated mean phastCons scores for refGene exon regions, refGene intron regions, and lincRNA control set. For each lincRNA transcript, the structure matched regions with same length and exonic structure was randomly sampled from unannotated regions of the genome, but regions within 10 kb of any annotated protein-coding genes were excluded. Eventually, the randomly generated list of transcripts was used as the control set for lincRNAs in the study. A cumulative frequency was plotted based on the distribution of phastCons scores.

### Syntenic orthologs of chicken lincRNA in human and mouse

TransMap orientates genes and related annotations in one species to another using synteny-filtered BLASTZ alignment to determine the most likely orthologs. Based on TransMap information, chicken orthologous genes in human and mouse can be identified. To find syntenic orthologs for chicken lincRNAs, we firstly determined orthologous genes in human and mouse for the lincRNA adjacent protein-coding genes. If both neighboring protein-coding genes of a lincRNA were found corresponding orthologs in human or mouse, we checked whether the orthologous gene pair was also adjacent to conserved relative orientation in the human or mouse genome. If they were positionally equivalent, non-coding transcripts annotated between them (if any) were considered as candidate syntenic orthologs for chicken lincRNAs in human and mouse. Only lincRNAs on autosomes were included in this analysis.

### Correlation matrix between lincRNAs and protein-coding genes

We used both refGene and ensGene annotations to identify nearest 3′ and 5′ neighbors of lincRNAs and RefGene annotation was used preferentially when the nearest protein-coding gene was annotated in both of refGene and ensGene annotations. Ensembl gene annotation was used when the distance from the ensemble gene to the lincRNA locus was smaller than the distance from nearest refGene annotation. Pearson correlation coefficients were calculated for each lincRNA and its neighboring protein-coding gene pair. As controls, we also generated neighboring protein-protein gene pairs and random protein-coding gene pairs by random sampling. As described above, the Pearson correlation coefficients were used for the two control sets. Finally, Kolmogorov-Smirnov test was applied to compare the strength of expression correlation.

To obtain the profile of functional correlation between lincRNAs and annotated genes, a correlation matrix of expressions was generated across 12 groups for each pair of lincRNA and protein-coding gene. Next, Gene Set Enrichment Analysis (GSEA)^64^ was applied for protein-coding genes with ranked Pearson correlation coefficient for each lincRNA and significantly enriched functional gene sets were defined by GO terms. The matrix was first clustered by GO gene set and then hierarchically clustered by lincRNAs. GO gene sets were clustered into 8 distinct functional groups with gene sets in each group sharing similar annotations. A heatmap was generated based on the clustered correlation matrix.

### Experimental validation of lincRNAs

The protocols of mRNA extraction and dsDNA synthesis were the same as those mentioned above. Real-time PCR using SYBR Green PCR Kit was utilized to validate multiple unannotated transcripts coupled with the conventional PCR based on iCycler iQ PCR System (Bio-Rad). The primers for conventional PCR amplification were designed using Primer3 (http://fokker.wi.mit.edu/primer3/input.htm) and confirmed by Oligo 6.0. The detail information about the primers we used is listed in Supplementary Table 10-11. The melting temperatures were between 55 and 65 °C and the length of the amplicons was between 80–500 bp. These primer pairs were designed to span over exons based on chicken genome. Chicken genomic DNA was used as control in PCR amplification. The primers for real-time PCR assay were designed using Oligo 6.0. The melting temperature was set at 60 °C and the length of the amplicons was between 50–200 bp. The primer pairs were designed within exons. Replicates were performed for RT-qPCR reactions. qPCR reaction was run using the program as follow: pre-incubation (95 °C for 10 min), 40 cycles of amplification (95 °C for 10 s, 60 °C for 10 s, and 72 °C for 10 s), melting curves using a heat ramp and cool down. Cycle threshold values (Ct values) were obtained from iCycler iQ PCR software. The expressions of lincRNAs and genes were normalized against *GAPDH* cDNA in the corresponding samples. The relative fold enrichment of each treatment group was calculated by comparing the enrichment value for the given primer pair to *GAPDH*.

## Additional Information

**How to cite this article**: He, Y. *et al.* The conservation and signatures of lincRNAs in Marek's disease of chicken. *Sci. Rep.* **5**, 15184; doi: 10.1038/srep15184 (2015).

## Supplementary Material

Supplementary dataset 1

Supplementary dataset 2

Supplementary dataset 3

Supplementary Information
