# Corrigendum: The conservation and signatures of lincRNAs in Marek’s disease of chicken

**DOI:** 10.1038/srep19422

**Published:** 2016-01-27

**Authors:** Yanghua He, Yi Ding, Fei Zhan, Huanmin Zhang, Bo Han, Gangqing Hu, Keji Zhao, Ning Yang, Ying Yu, Li Mao, Jiuzhou Song

Scientific Reports 5: Article number: 15184; 10.1038/srep15184published online 10162015; updated: 01272016

There is an error in the Acknowledgments section of this Article.

“This project was supported by the National Research Initiative Competitive Grant (No. USDA-NRI/NIFA 2010-65205-20588) from the USDA National Institute of Food and Agriculture and by the China National Science Foundations International Program Competitive Grant (No. 21023130).”

should read:

“This project was supported by the National Research Initiative Competitive Grant (No. USDA-NRI/NIFA 2010-65205-20588) from the USDA National Institute of Food and Agriculture and by the National Natural Science Foundation of China (No. 31320103905).”

