# Supplementary material for: The conservation and signatures of lincRNAs in Marek’s disease of chicken
Source: Sci Rep. 2015 Oct 16;5:15184. doi: 10.1038/srep15184 (PMC4608010; doi:10.1038/srep15184)
Supplement: Supplementary Information [file srep15184-s4.pdf]

The conservation and signatures of lincRNAs in Marek's disease of chicken

Yanghua He[1*], Yi Ding[1*], Fei Zhan[1*], Huanmin Zhang[2], Bo Han[4], Gangqing Hu[3], Keji Zhao[3], Ning Yang[4], Ying Yu[4], Li Mao[5], Jiuzhou Song[1§]

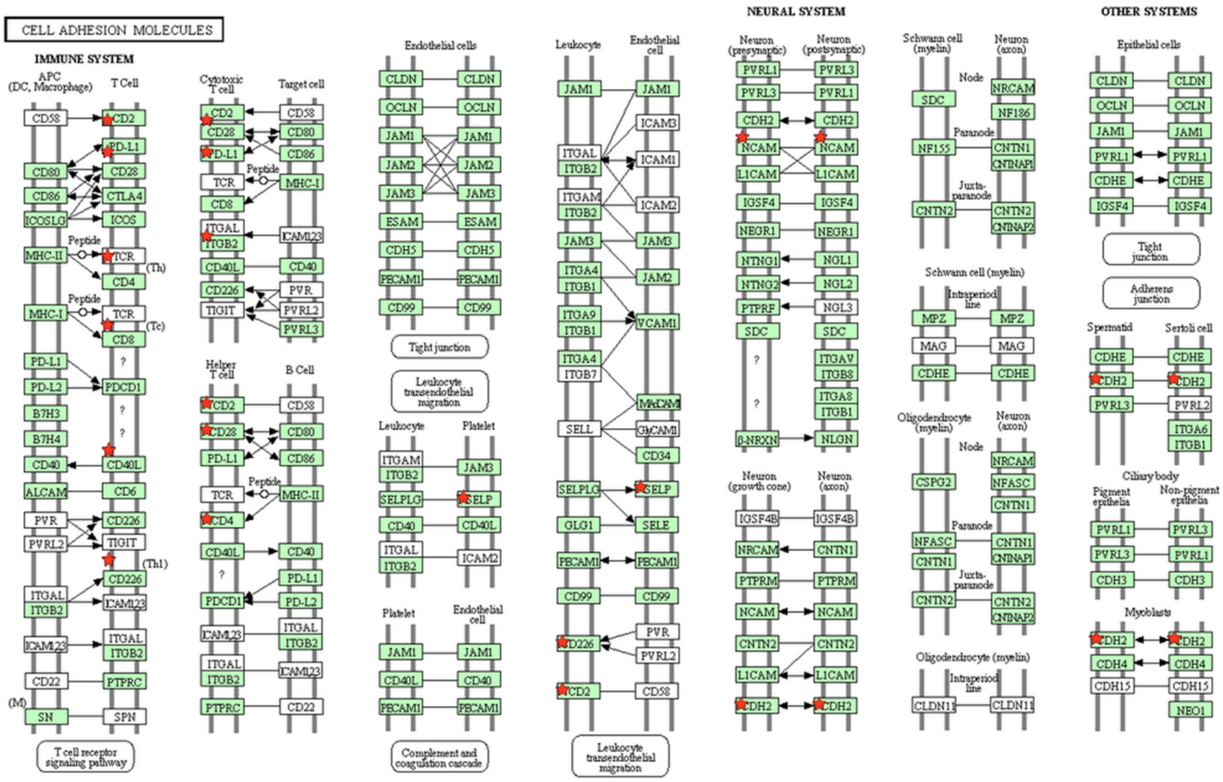

Supplementary Figure 1 | Cell adhesion molecules pathway involving by up-regulated differential expressed genes after MDV infection in line 6_3. List genes are shown in red star.

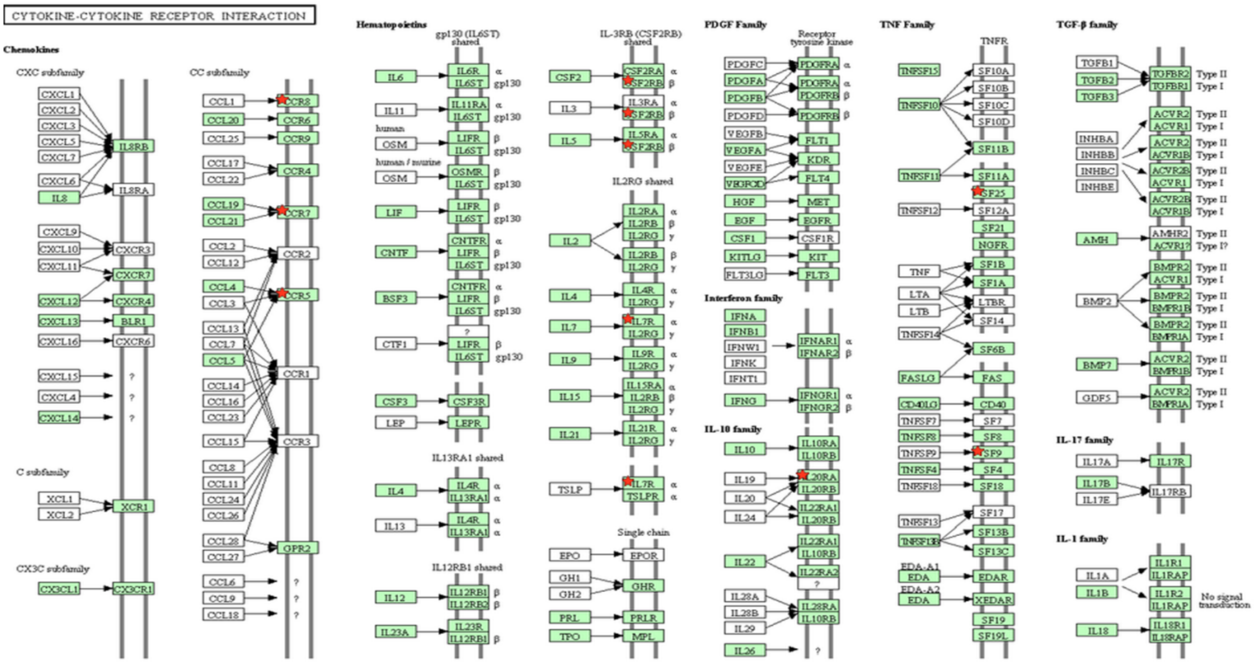

Supplementary Figure 2 | Cytokine-cytokine receptor interaction pathway involving by up-regulated differential expressed genes after MDV infection in line 6_3. List genes are shown in red star.

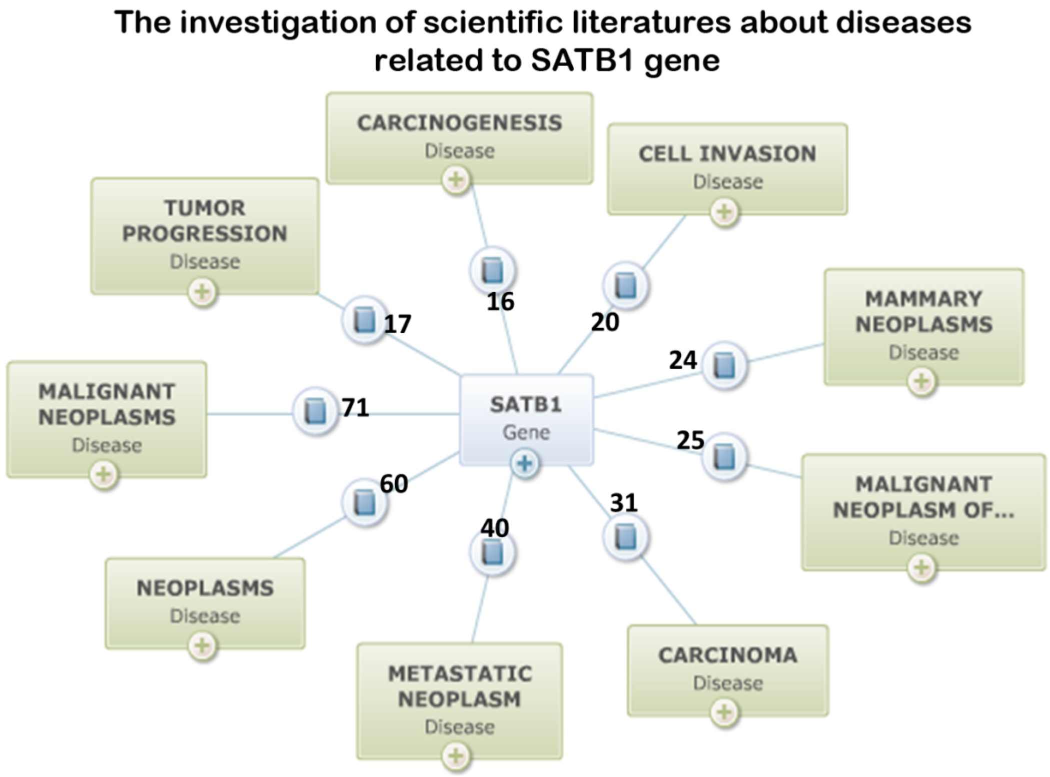

Supplementary Figure 3 | The investigation of scientific literatures about diseases related to *SATB1* gene. The number indicates number of publications corresponding to this particular disease up to now.

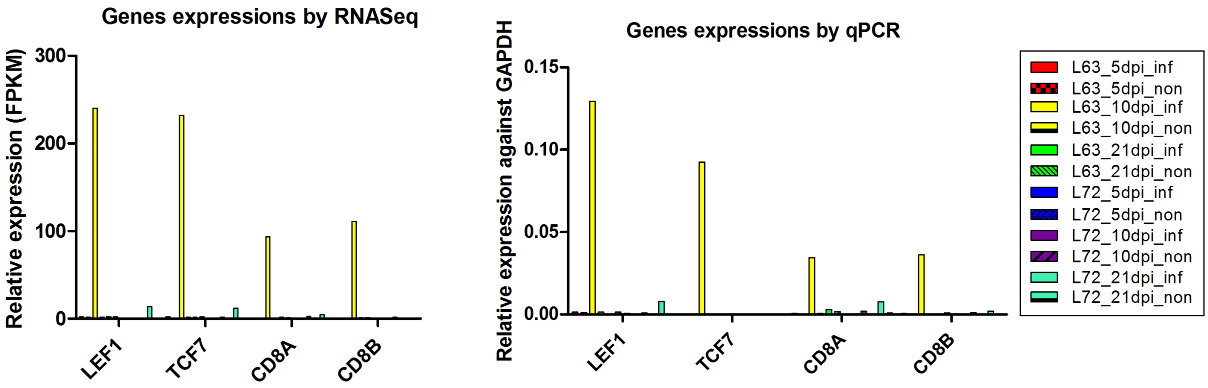

Supplementary Figure 4 | The validation of expression about *SATB1* related genes by qPCR experiments. *LEF1* and *TCF7* as T lymphocyte associated activators as well as cytotoxic T cell co-receptor *CD8A* and *CD8B* were chosen to validate their expressions across two conditions of two chicken lines at three time points.

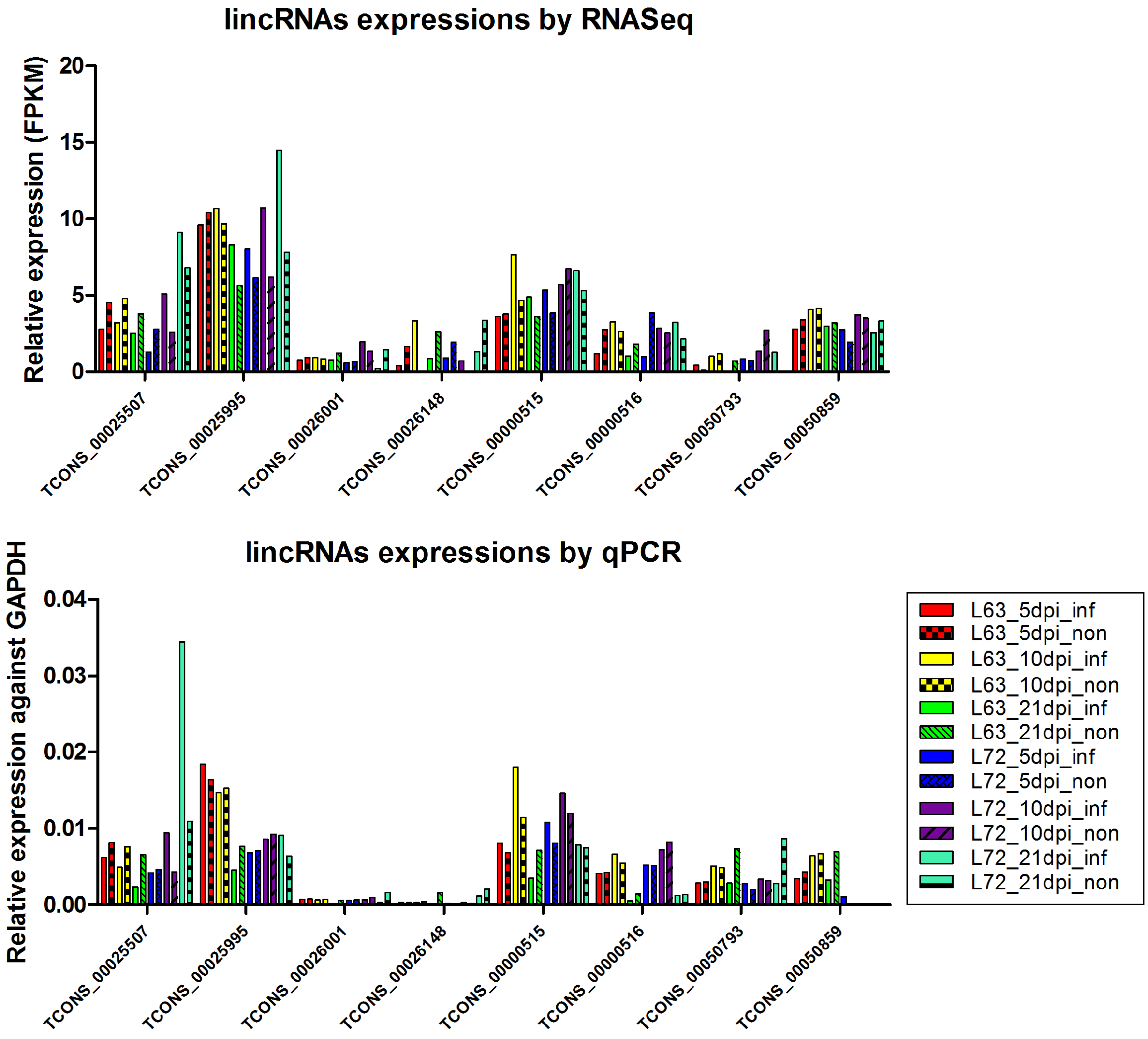

Supplementary Figure 5 | The validation of candidate lincRNAs expressions by qPCR experiments in various treatment conditions. The expression levels were normalized against *GAPDH* cDNA in the corresponding samples.

Supplementary Table 1 | Mapping statistics of sequencing reads

Line	DPI	Treatment	Raw reads	Mapped reads	Unmapped reads	Percent mapped
L63	5dpi	Infected	26,436,718	22,757,281	3,679,437	86.08%
			25,362,239	21,849,245	3,512,994	86.15%
		Non-infected	20,830,331	13,103,489	7,726,842	62.91%
			20,217,983	14,613,688	5,604,295	72.28%
	10dpi	Infected	32,070,953	27,650,629	4,420,324	86.22%
			27,656,512	23,698,520	3,957,992	85.69%
		Non-infected	19,643,972	11,361,656	8,282,316	57.84%
			17,741,686	11,756,499	5,985,187	66.26%
	21dpi	Infected	24,764,470	19,245,364	5,519,106	77.71%
			16,377,980	14,098,784	2,279,196	86.08%
		Non-infected	23,120,214	17,400,805	5,719,409	75.26%
			12,829,650	9,403,356	3,426,294	73.29%
L72	5dpi	Infected	27,855,862	19,824,345	8,031,517	71.17%
			31,178,311	20,499,964	10,678,347	65.75%
		Non-infected	33,818,194	21,314,161	12,504,033	63.03%
			31,334,208	20,067,622	11,266,586	64.04%
	10dpi	Infected	21,258,758	13,979,594	7,279,164	65.76%
			20,030,690	13,809,847	6,220,843	68.94%
		Non-infected	24,005,256	16,038,502	7,966,754	66.81%
			28,146,097	19,191,739	8,954,358	68.19%
	21dpi	Infected	30,141,835	15,226,575	14,915,260	50.52%
			29,783,547	12,667,613	17,115,934	42.53%
		Non-infected	24,061,054	15,483,473	8,577,581	64.35%
			29,227,316	21,234,014	7,993,302	72.65%

Supplementary Table 2 | Enriched GO terms for lincRNA neighboring protein-coding genes

Term	Description	Number of neighboring genes	P-value
GO:0042981	regulation of apoptosis	19	0.000991
GO:0001568	blood vessel development	11	0.002544
GO:0045449	regulation of transcription	44	0.031890
GO:0032268	regulation of cellular protein metabolic process	12	0.004934
GO:0006796	phosphate metabolic process	30	0.027536

Supplementary Table 3 | Pathways of differential upregulated genes by MDV infection in line 6_3

**Cell adhesion molecules (CAMs) pathway**						
ID	ENSEMBL_GENE_ID	GENE NAME	L63_10dpi_inf_FPKM	L63_10dpi_non_FPKM	L72_21dpi_inf_FPKM	L72_21dpi_non_FPKM
1	ENSGALG00000015463	CD2 molecule	21.0648	4.48068	NS	NS
2	ENSGALG00000013748	CD226 molecule	14.4446	1.48143	NS	NS
3	ENSGALG00000008669	CD28 molecule	68.076	1.20588	16.0991	2.75903
4	ENSGALG00000014477	CD4 molecule	54.2343	1.0946	23.5649	0.42153
5	ENSGALG00000015816	CD8a molecule	91.0804	0.796491	NS	NS
6	ENSGALG00000015902	CD8b molecule	102.911	0.974445	NS	NS
7	ENSGALG00000015132	cadherin 2, type 1, N-cadherin (neuronal)	5.25906	1.05938	NS	NS
8	ENSGALG00000008656	inducible T-cell co-stimulator	33.1506	2.58348	24.2536	2.56503
9	ENSGALG00000002643	selectin P (granule membrane protein 140kDa, antigen CD62)	12.7475	3.08303	NS	NS
**Cytokine-cytokine receptor interaction pathway**						
1	ENSGALG00000011733	chemokine (C-C motif) receptor 2	11.8788	3.18372	45.4437	3.87589
2	ENSGALG00000003888	chemokine (C-C motif) receptor 7	41.996	6.08314	NS	NS
3	ENSGALG00000011954	chemokine (C-C motif) receptor 8	4.46522	0.709297	27.1077	0.551175
4	ENSGALG00000011734	chemokine (C-C motif) receptor 8	3.84244	0.323736	9.59802	0.827927
5	ENSGALG00000012518	colony stimulating factor 2 receptor, beta, low-affinity (granulocyte-macrophage)	7.44588	1.743	NS	NS
6	ENSGALG00000013869	interleukin 20 receptor, alpha	17.0242	4.63652	23.5236	3.78336
7	ENSGALG00000013372	interleukin 7 receptor	16.098	3.21868	10.2675	1.64534
8	ENSGALG00000017614	similar to death receptor 3	40.0386	5.33619	NS	NS
9	ENSGALG00000000543	tumor necrosis factor receptor superfamily, member 9	47.8573	14.7241	57.6137	12.1837

Note: 'NS' represents no significant difference between infected and non-infected chickens (FDR > 0.05).

Supplementary Table 4 | Fold changes of lincRNAs expressions between infected and non-infected chickens across two lines and three time points as well as their nearest gene associated with immune response

	lincRNAs	L63_5 dpi	L63_10 dpi	L63_21 dpi	L72_5 dpi	L72_10 dpi	L72_21 dpi	Transcript	Nearest gene	Distance
1	XLOC_068940	-0.038	2.112	0.253	-0.589	-0.758	0.406	TCONS_00070365	ENSGALG00000005543	8002
2	XLOC_000789	0.059	1.577	0.000	-0.649	-0.074	0.342	TCONS_00001017	ENSGALG00000015494	9930
3	XLOC_063601	0.496	2.229	-0.448	-1.070	-0.852	0.662	TCONS_00064948	ENSGALG00000008466	5242
4	XLOC_068939	0.067	2.985	-0.032	-0.777	-0.032	1.413	TCONS_00070364	ENSGALG00000005544	8730
5	XLOC_024972	-0.024	1.817	-0.022	-0.328	0.184	1.441	TCONS_00025556	NM_205396	7834
6	XLOC_024939	0.153	3.969	-0.205	-0.310	0.186	1.026	TCONS_00025519	NM_001199644	4335
7	XLOC_049658	0.156	1.767	0.146	-0.649	0.172	1.678	TCONS_00050808	ENSGALG00000016038	74670
8	XLOC_071992	0.314	1.052	0.029	-1.244	0.711	1.720	TCONS_00073753	NM_204604	61542
9	XLOC_022076	0.403	0.556	-0.361	-0.742	0.636	1.321	TCONS_00022602	ENSGALG00000002982	559
10	XLOC_022110	0.204	1.155	0.111	-2.478	0.650	1.761	TCONS_00022640	ENSGALG00000003677	17286
11	XLOC_075869	0.256	0.135	0.073	-2.010	-0.830	0.846	TCONS_00078338	ENSGALG00000000273	1460
12	XLOC_075809	-0.448	1.293	-1.206	-2.014	0.514	0.398	TCONS_00078256	ENSGALG00000014714	1700
13	XLOC_025293	0.125	0.761	-0.129	-0.439	0.195	-0.338	TCONS_00025976	ENSGALG00000017395	1234
14	XLOC_035285	0.171	0.148	0.280	-0.118	-0.631	0.164	TCONS_00036079	ENSGALG00000007250	1708
15	XLOC_075868	-0.267	0.712	0.297	-1.529	0.130	0.745	TCONS_00078337	ENSGALG00000000264	4272
16	XLOC_056	0.359	1.903	0.507	-0.690	0.773	-0.203	TCONS_000	NM_204801	641

	648							57885		
17	XLOC_068949	-0.019	1.799	-0.004	-0.263	0.141	-0.180	TCONS_00070375	ENSGALG00000005118	9309
18	XLOC_041314	0.422	0.905	0.791	-0.467	0.212	-0.160	TCONS_00042404	ENSGALG00000022887	1916
19	XLOC_000726	0.000	1.062	0.518	-0.463	0.000	0.000	TCONS_00000927	ENSGALG00000017280	1620
20	XLOC_066491	-0.009	3.031	-0.250	-0.417	-0.345	-0.011	TCONS_00067888	ENSGALG00000002182	78152
21	XLOC_056530	0.031	1.072	0.025	-0.661	0.062	0.080	TCONS_00057745	NM_001012585	20746
22	XLOC_015830	0.000	2.092	-0.516	-0.149	0.189	-0.190	TCONS_00016228	ENSGALG00000020778	12608
23	XLOC_072297	0.857	0.783	-0.017	-0.428	0.000	1.904	TCONS_00074211	ENSGALG00000003238	42910
24	XLOC_022092	-0.065	0.434	-0.131	-0.261	0.527	1.027	TCONS_00022620	ENSGALG00000008886	973
25	XLOC_049655	0.773	2.079	-1.621	-0.634	-0.283	2.763	TCONS_00050802	NM_204991	60541
26	XLOC_017708	0.989	0.624	1.874	-0.616	0.410	2.580	TCONS_00018157	ENSGALG00000007620	2357
27	XLOC_019436	0.097	0.487	0.491	0.311	0.239	0.504	TCONS_00019915	ENSGALG00000004460	140
28	XLOC_025407	-0.659	0.401	-0.210	0.110	2.180	0.803	TCONS_00026130	ENSGALG00000012741	64086
29	XLOC_073134	0.210	0.529	-0.581	-0.609	-0.068	0.294	TCONS_00075433	ENSGALG00000022091	5043
30	XLOC_025574	0.049	-0.466	-0.379	0.449	0.968	-0.822	TCONS_00026325	NM_001012555	3003

Supplementary Table 5 | Identifying the initiation codons in linc-satb1 sequence by Prediction of Translation Initiation ATG (http://atgpr.dbcls.jp/)

No.of ATG from 5'end[1]	Reliability[2]	Frame	Identity to Kozak rule A/GXXATGG	Start (bp)	Finish (bp)	ORF Length (aa)	Stop codon found?	Sequence
46	0.09	2	GXXATGc	2333	2491	53	Yes	MLLWHNDLEISFCVRRAIGSSGKHYRYFPVPFRHVNMFRHEQRVELGSGVGWG
9	0.07	2	GXXATGt	689	826	46	Yes	MFTLLQSQLSAASSLSSSVCLTSMLLLSKTHLLYLYHTPVWLVSES
77	0.07	2	cXXATGc	3362	3454	31	Yes	MLHSTSIHSASLQDGNYFPLHISAAEDLVIT
80	0.07	2	GXXATGt	3539	3706	56	Yes	MSDSLLCCLIALSTFCQSETLILERTEGVVLIFVLSTFFLHIVCSFPWCLVCLGCF
57	0.06	2	cXXATGa	2636	2776	47	Yes	MIKREGPLLLFITNDNRTLHNAQMLASIPRNANFMKYDLFPSQSVQL

[1]No. of ATG from 5' end: A sequence may have many ATG triplets. This information tells you the number of an ATG's occurrence relative to the 5' end

[2]Reliability: For example, a reliability of 0.12 means that in our tests the reliability of the ldf score was only 12% and should therefore be treated with the upmost caution.

Supplementary Table 6 | Gene Set Enrichment Analysis of target genes of *SATB1*

	HAN_SATB1_TARGETS_UP (193*)			HAN_SATB1_TARGETS_DN (224*)		
	Inf vs. Non	6_3 vs. 7_2	10dpi vs. rest	Inf vs. Non	6_3 vs. 7_2	10dpi vs. rest
Number of significant enrichment genes	95	37	92	106	55	73
Percent of significant enrichment genes	49.2%	19.2%	47.7%	47.3%	24.6%	32.6%

*represents the total number of orthologous genes in chicken matched to Gene sets of HAN_SATB1_TARGETS (Homo sapiens).

Supplementary Table 7 | The transcriptional enhancers in the human and mouse genomes by Vista Enhancer Browser (http://enhancer.lbl.gov)

ID	Human (hg19)		Mouse (mm9)		Expression
	Coordinates	Bracketing Genes	Coordinates	Bracketing Genes	
hs250	chr3:18,169,362-18,170,237	LOC339862(intragenic)	chr17:51,675,683-51,676,643	C330011F03-Satb1	Positive
hs251	chr3:18,844,368-18,845,392	SATB1-KCNH8	chr17:52,364,835-52,365,855	Gm20098-Kcnh8	Negative
hs651	chr3:18,898,614-18,899,860	SATB1-KCNH8	chr17:52,447,155-52,448,401	Gm20098-Kcnh8	Negative
hs825	chr3:18,916,569-18,917,473	SATB1-KCNH8	chr17:52,469,177-52,470,077	Gm20098-Kcnh8	Negative
hs936	chr3:19,027,747-19,029,355	SATB1-KCNH8	chr17:52,598,809-52,600,417	Gm20098-Kcnh8	Negative
hs252	chr3:19,033,849-19,034,569	SATB1-KCNH8	chr17:52,604,445-52,605,152	Gm20098-Kcnh8	Negative

Note:

Positive indicates that this sequence showed observable enhancer activities at E11.5.

Negative indicates that this sequence did not show observable enhancer activities at E11.5 and it was a negative enhancer at this developmental stage. This does not mean that this sequence is not an enhancer at another time point or a weak enhancer at this time point.

Supplementary Table 8 | The coordinates of *SATB1* and *KCNH8* genes as well as non-coding RNA in human, mouse and chicken genomes

Genes/ncRNAs	Human (hg19)	Mouse (mm9)	Chicken (galGal3)
SATB1	Chr3: 18389133-18466829	Chr17: 51875512-51971972	Chr2: 35135980-35226779
ncRNA	Chr3:18,844,368-19,034,569	Chr17: 52201562-52201871	Chr2: 35231116-35236359
KCNH8	Chr3: 19190017-19577135	Chr17: 52742034-53044544	Chr2: 35538119-35650436

Note: ncRNAs for human were showed in Table, ncRNA for mouse is GM20098/NR_045095, and for chicken is *linc-satb1*.

Supplementary Table 9 | LincRNA as precursors for small regulatory RNAs

Target name	Accession	Query name	Description
**microRNAs**			
mir-684	RF00876	TCONS_00000218	microRNA mir-684
mir-207	RF00802	TCONS_00000218	microRNA mir-207
mir-279	RF00754	TCONS_00012819	microRNA mir-279
MIR828	RF01026	TCONS_00078633	microRNA MIR828
mir-573	RF01040	TCONS_00025556	microRNA mir-573
miR-430	RF01413	TCONS_00038360	microRNA miR-430
mir-279	RF00754	TCONS_00064993	microRNA mir-279
mir-207	RF00802	TCONS_00074891	microRNA mir-207
**snoRNAs**			
TB11Cs4H1	RF01539	TCONS_00001014	Trypanosomatid snoRNA TB11Cs4H1
TB11Cs4H1	RF01539	TCONS_00000421	Trypanosomatid snoRNA TB11Cs4H1
sR11	RF01150	TCONS_00014387	Small nucleolar RNA sR11
snoZ152	RF00350	TCONS_00014387	Small nucleolar RNA Z152/R70/R12/
ceN47	RF01637	TCONS_00057778	C. elegans snoRNA ceN47
SNORA55	RF00431	TCONS_00074562	Small nucleolar RNA SNORA55
**telomerase RNAs**			
Telomerase-vert	RF00024	TCONS_00074562	Vertebrate telomerase RNA
Telomerase-vert	RF00024	TCONS_00074562	Vertebrate telomerase RNA

Supplementary Table 10 | Primers used to confirm lincRNAs structure

PCR	Primer name	Sequence (5’ → 3’)	Product Length (bp)	Template	Annealing temperature (°C)
XLOC_038005	S05	F: 5’ CTGAGCAAGGACGTAGAGA 3’<br>R: 5’ CAGAGATGCTGTGACGTTT 3’	188	dscDNA	63
XLOC_017676	S76	F: 5’ AGCTTTCATAGGGGTTTCA 3’<br>R: 5’ GGAGCTCTTATGAAGGCTG 3’	179	dscDNA	61
XLOC_035296	S96	F: 5’ ACAGTTGCACACCGTGTTTC3’<br>R: 5’ GTGTCCTTTCCATGCCTGTT 3’	194	dscDNA	63
XLOC_041037	S37	F: 5’ TTAACATCCAAGCAGCTAC 3’<br>R: 5’ TTTTCCAGATGTTGATGTT 3’	480	dscDNA	63
XLOC_024939	linc-satb1	F: 5' GCTGCTCCTTTCAGTGCATT 3'<br>R: 5' CTGACATTGCTGACGCATCT 3'	333	dscDNA	60

Supplementary Table 11 | Primers used to confirm lincRNAs and genes expression levels

qPCR	Primer name	Sequence (5’ → 3’)	Product Length (bp)	Template	Annealing temperature (°C)
Linc-SATB1	Q39	F: 5' GGTGTAGTTGTAGGCCAGCA 3'<br>R: 5' TGCACGCCAGGTAAGCAATA 3'	80	dscDNA	60
TCONS_00025507	HOTTIP	F: 5' GGGGAATAAAACAACGAGACAGA 3'<br>R: 5' AGAACCATCGCACGTAGAGG 3'	120	dscDNA	60
XLOC_000566	Q66	F: 5'GGCTTTCTCTGGGAGCTGTT 3'<br>R: 5'GACCTCTGGCTTTTCTCCCC 3'	127	dscDNA	60
XLOC_039595	Q89	F: 5' ATGACCGTGGATACAACA 3'<br>R: 5' GCACAGAGGTCGGTTAT 3'	55	dscDNA	60
XLOC_041037	Q41	F: 5' GGACTGATTACTTGGGCCCC 3'<br>R: 5' CCATCGCCTTCCCTTCTGTT 3'	67	dscDNA	60
XLOC_000866	Q06	F: 5' GCACTACCCTGCCAAGTGAA 3'	152	dscDNA	60

		R: 5' GGTTGCAACTGTGAGGCTTG 3'			
XLOC_041175	Q26	F: 5' TGAGGCCCTTGTAATGCCAG 3'<br>R: 5' CTAGTCACCCTTCTGCCTGC 3'	122	dscDNA	60
XLOC_025339	Q30	F: 5' TAAGCTACACCGCCACACAG 3'<br>R: 5' TCCCTGGACCACATCAGACT 3'	162	dscDNA	60
TCONS_00025995	Q95	F: 5' ACATCTCCCTTCTAGCCTGGT 3'<br>R: 5' AAGTTCACAGCTGGCTCTCC 3'	140	dscDNA	60
TCONS_00026001	Q01	F: 5' TCTCCCTCCCCAGACTGAAG 3'<br>R: 5' GGAAGGAATTGCAGCGCTTC 3'	111	dscDNA	60
TCONS_00026148	Q48	F: 5' TTCCCAAGAACGATGCTGCT 3'<br>R: 5' CAATCGATGGGCTGGTTGTG 3'	104	dscDNA	60
TCONS_00000515	Q15	F: 5' CTCCATGTGCATCCCTTGGT 3'<br>R: 5' GCATCGTCGCTTTAGTCACAC 3'	126	dscDNA	60
TCONS_00000516	Q16	F: 5' GTCTGCGGAATCAAGGGACA 3'<br>R: 5' CACCTTCACCATGGCAGTCC 3'	189	dscDNA	60
TCONS_00050793	Q93	F: 5' CAGACCATAAGCGCTGTCCA 3'<br>R: 5' TGCTGGATGTATTGGCAGCA 3'	90	dscDNA	60
TCONS_00050859	Q59	F: 5' CACCCTTAGCAAGTCCTGGG 3'<br>R: 5' CTCTAGTACAGCGCCACCAG 3'	104	dscDNA	60
SATB1	SA-1	F: 5' AGCCCAGCAGTCCTTAAACC 3'<br>R: 5' ACTGCCTGTGAGATTCCTGC 3'	165	dscDNA	60
HOXA2	HA-2	F: 5' TGCGAGTTGAAGGCCATGAA 3'<br>R: 5' AGTGTCGAGTGTGAAAGCGT 3'	155	dscDNA	60
HOXA3	HA-3	F: 5' GCGACCTACTACGACAGCTC 3'<br>R: 5' GCGGTGGTACTCAGTTTCCA 3'	129	dscDNA	60
HOXA4	HA-4	F: 5' TGAAACCAAAACCCGGACGA 3'<br>R: 5' GAGGGCAAGAGAGGGTTTCC 3'	96	dscDNA	60
HOXA9	HA-9	F: 5' CGCTGGAACTGGAGAAGGAG 3'<br>R: 5' CCCCGTATCCCCATCATTCG 3'	180	dscDNA	60

HOXA10	HA-10	F: 5' CTACGTGTCGGGGATGGAAG 3'<br>R: 5' CCTTGGGGCACTTCTCTGAG 3'	152	dscDNA	60
HOXA11	HA-11	F: 5' CCGTCTTCTCGCCCTATGAC 3'<br>R: 5' GTGGAAGGCAAGCAGTCTCT 3'	179	dscDNA	60
HOXA13	HA-13	F: 5' TCGCCGACAAGTACATGGAC 3'<br>R: 5' CCTGGTAGAAGGCGAACTCC 3'	78	dscDNA	60
LEF1	LF-1	F: 5' CTACCATGACAAGGGCAGGG 3'<br>R: 5' GATGTTCTGGGGATGGGTGG 3'	171	dscDNA	60
TCF7	TF-7	F: 5' AAGCAGCAGGACTCCAACTC 3'<br>R: 5' GTCCGTTTGTTGGTTGAGGC 3'	78	dscDNA	60
CD8A	C8-A	F: 5' AAAGGCCATCTCCCACACAG 3'<br>R: 5' CGGTGTCACTCTGCTCCTTT 3'	86	dscDNA	60
CD8B	C8-B	F: 5' CAGCCAGGAGAAGTTCAGCA 3'<br>R: 5' ATGAGCTGGGAGGACTGGAT 3'	126	dscDNA	60
GAPDH	HK-1	F: 5'-GAGGGTAGTGAAGGCTGCTG-3'<br>R: 5'-ACCAGGAAACAAGCTTGACG-3'	307	dscDNA	60

The sequence of linc-satb1:

TCONS_00025519

>galGal3_ct_UserTrack_3545_TCONS_00025519_0 range=chr2:35231116-35232293 5'pad=0 3'pad=0 strand=+ repeatMasking=noneCTCTTTCTCTCTTTCTTTTTTTCCCCTTCTTTTTATCTTTTCCTTTATTTTGACTTTGAG
GTGCATTTTCTCTTCCTGCTCTGGGAATTGGCAGATACAGTTCCTGTAGGCAACTTTGCATCAAGACAG
AGACTGTGAAGTTGATACCTCAGTGCTTTTCTTAGCTGTGCTCTTCTATTAATTGGTGTTAGAAAGTGC
AACCTTGTAGCACAGTTATTGAATAATCATTAATCTGTGGAGGATACACAAGCCCAAAGGTTAATAAT
AACAATTAATAATAGTGACTTTGCTAGCAGGGAGACTATTTTCTCATAGTTATTAAAAGGCACTCCCAT
TTATGCTATGTATGGAGAGGTGAATTTGGCCCTGGTGTGCTGAATACCACTTTACCGCAGCAAGCCCA
CAGATGAATCCTCCCCATTATCTCCATGCTGTCAGGTCTCTCGCTTTTTCTCCCTTTAAAATGTATCAAA
CACTGCTCTCATTTTAAATAGAAATGCTCAGGCTTTAATCAATTCTGACATTTCCACTGGAAAATAACA
TCTAGCTCTCTTACACAGATAAAAATTTATTTGCCTGCAGCTGGTCTGCTGTAGCGCTTTGGACCTATT
CGCTTCTCAGAGAAGACTTGGCTTTTCATGGTCTCTAGGTACCACGGGGCAATAAATAGAAAGTCAGT
GCAAGGGGGATGTTCACACTTTTACAGTCACAACTCTCCGCTGCCTCCTCGCTGAGCTCTTCTGTCTGC
CTAACCAGCATGCTCCTGTTGTCCAAGACCCATCTCCTTTATTTGTATCACACCCCTGTCTGGCTGGTCA
GTGAATCTTAATAATAATCATGTTTCATGTCTCAGAAATCTAATAATCAAGTTACTCATTCGTATAAAT
TGCAACTAAGCTGTTTGGATTCGTTGTGTTATCCCCACTTTAAAGGCACGGAAACTGAAACAGAAAAA
TTAACACATTTGCCTGAGGCCACACATGGATTGAAGAGTATGTCCAGCACTGGAACTGAATTCTGTAT
TTAGACCATGCTTCCTCTTAAAGATTGTGAAGCACACTGTGGAAAATGTTTTAAAGAAAGGAATGTA
GTATAAACCTTATTTTCCATTGACTTTGTATACTAGTCAAATTGCATGCCTTTTTTTTTTTTTGGGGGGG
GGGGGGGGGGGGGGGAG

>galGal3_ct_UserTrack_3545_TCONS_00025519_1 range=chr2:35232838-35234977 5'pad=0 3'pad=0 strand=+ repeatMasking=noneGGGGGGGGGGGGAGGTTTAATATATATCACGTTTCCTTTTTTAAATACAAGATTTAT
TTGACTTTTGACAAAGCACGGTGGAACCTTTCAGTTGCAGTCAGCTTACTGTGGAAACAAGAAACATT
TACTGTGAGATTGTGAGCAGTAATGGGAACAGAATTGCTTGTTATTAGGTTATTGTAAACAGTATCCAT
TGGATGTTACGGTGTAGTTGTAGGCCAGCACAATATAATGAGAAGTTCAGCACTAGTACAGATAAGGT
GCTATTGCTTACCTGGCGTGCATCAGTTTCCACCGTAATTCTCCTTCAGTGCTTTCAGTAGCAAAATAC
TTAGTGTGGGTTTAAGCACTGAAAAACATGTGGGACAGCCATATACTGTGAGAGTTATGATTTATATC
ATTAAGCAGTGAGTCTTGCAGTGTTGTGTCACTCATGTCAATGAAGCAAGAAGTATACTGCCGGCCCT
TTAGCTGTGGCATGTCATCAAAGCTATATTATGGTATGACTGAGCACAGATGGCATGTCTAAAAAGCA
GGTGTACAATATAACTTTCATATTACTGACACGTAGATATTTCCTCTTCCTCAGTGTCCATTCCCAGAT
GTTTGCTTGCCCAACCTTCTACAGGAACTAAAGAAAGTCCTGCTCATCCTGCTCATCCTTCATGTCTGG
AATTGTATCATCAAAACCCATAGAGAATATGTGTTTATATATATTTGTATGTATTTATGCTGACAGCTC
CTTTCTGAGTTTTTTTTCAGAGTCCTGATTTGAGGAAAGTGGATGCAGATCTCACTCAAATAATCTTTCT
CCAATACTATCAACATTGCCTGCCAAAAATAAAAGTGGCATATGAATCACAATAATTCACTCATGATT
TGGTGGATTTAAAGCCTGCGAAGGAGCATTAGAAATCCACATGGTGCTCCTGAGCTTTATGGACTTTG
AATGGTGGTAAAAGTATATTTAGCCTAAGACTATCTTATTGATGATCTGTCTTATATGGGCTGCAGATC
TATCACCTATTCAAGTGAAGGACAATATTAATTGAAGCTATCTCTGTCCAAATGTTTATCTTTCAGGTG
AGGTAATGAAACTGTTGAATCTGCAGACTGAAACCGTCAGAAGCCTTTCCTGAGGTGCCCCTTACTGA
AATGCTACTGTGGCATAATGATTTGGAAATAAGCTTTTGTGTGAGAAGAGCTATCGGATCCTCTGGGA
AACATTACAGATACTTTCCAGTTCCTTTCAGACATGTAAATATGTTTAGACATGAGCAGAGGGTAGAG
CTGGGGAGTGGAGTAGGGTGGGGTTAGAAGCTTCCTCATAGATCCTGGAACCAAATAAATACATGCGT
TTTTTTTTCTTCTTATCTGATGTTAGCTGGAACTTGAAATGGTCAATATTGAGAAGAAAAACTGCATAA
ATGTGTGTATATGTAAGAGTTATGTTGACCCATGATTAAAAGAGAGGGTCCTCTTCTGCTGTTCATCAC
AAATGATAACAGAACTCTCCATAATGCTCAGATGCTTGCATCGATCCCAAGAAATGCGAACTTTATGA
AGTATGACTTGTTTCCGTCTCAAAGTGTGCAGCTATAGCTATAGCACTGAAAACCGTTGCATGCTATCT

AGCATTTCTCATGATTTTTGCTTTCTTCATGATTTAAGCTAAGCACATCAGAAACTGAAGATAACGTTT
TAACCTATAAGAACAGCAGCCTGACTTCTGTAATGTGCTCATGTGCTATTACCTGTGAAACAGAGCAA
GAAGACTGCTCACTAGCAGGGTCTGCCCATCGCTGTCCAGCTGCCTAATGACATGTTAAGACTTGGTTT
CAGCAACTGTGCTCCAACCAGTATTACTATAATTGGTTGCAGTTGAAGATGTCAGTAAAAAAATACAA
TCAGGCTGGAGTGTCCTATGCATATTTTAAATGCAAATGGCCGTTATCAGCTGTATCATAACTTTATCT
GATGTCTGATCTTAGTGGTGGCTCTGTGGAAAAGACTCAGATGACTTTTTCTGAACACAGCTGCTCCTT
TCAGTGCATTTCTGCAGTCTTTTGATTATCTTACTTACTATCTGTTGGCTAGACACCAACCTCCTGAACT
TAGGCCTGTGTCACCTCACTACCTGT

>galGal3_ct_UserTrack_3545_TCONS_00025519_2 range=chr2:35235064-35236359 5'pad=0 3'pad=0 strand=+
repeatMasking=noneTTTCTTCCAGCCACTCACTGGGGGTGCTTGTTTCCAGGCCCTGATGCTGCACAGTAC
CTCAATCCATTCAGCTTCTCTTCAAGATGGTAACTACTTTCCGCTCCACATCTCTGCTGCAGAAGATCTT
GTGATTACCTGAAAAAGTGAATTGCATCCTGTAGATTTCCCCTTAAAACTTCTGCTTCACAGAAGAGAT
CAGCTTGTGACAGATGCGTCAGCAATGTCAGACTCACTTCTCTGTTGCCTAATTGCCCTCTCCACCTTC
TGTCAATCTGAAACTCTCATTTTGGAAAGGACTGAAGGAGTTGTATTAATATTTGTGCTATCCACATTT
TTCCTGCATATTGTCTGCTCCTTCCCTTGGTGTCTGGTTTGTCTTGGCTGCTTTTAAAAATTCTGTTGCCT
CTAGTGAGGGACTGCCAGATTAAACCAGTACAGAGAATCCAGGTGACTGTCTACTTTTACAAATATAG
GTTGTGTATAATAAATTCAGGTTTTACATCTATGCAAATTAAGTCATCATATGATTATGACTACATGCA
TTTTTTTATCAAATATGGCATATATTTCACAAATTATATTTAACTTATACACATGTTGCTATTCTGAATA
CCTGACTAGGAATTACAAAGTGTGTCACTAGATCTTAGTAAGTGGGCTTATGGAACAAGAAGAAATAA
GAGAAAATCACCTCTTTTCCTTGCATTCCTGACAAGTCCAGTGTAAGGGCTTTTCCTACTCTAGATGGC
AATATAAACACCTTACCCACAGAGCTGTTGAGAGATAAAAGTACATCTTGTTGTTGGAAACTTAAAGAA
TGTATGAAAAACAAAACAAAATACCCTAAAAAAGGAAGTTTTAAAGTAATGTGTCTTTGGTCTAGTGT
TTGTCACATCTACTTGGTATGGCCTTCCTCTAATGTTTCAGTCATGCAGTGTAAGACATTAGTCACAAT
GTCAAAATAAGCACATTACTTCTGCAAAAATGTATTTTCACCTCAGGAATATTGTGAAGTGAAAATAG
GATTTAGCACAATTCTTCCTTTTGAGTTTTTCTACAGACAGGACAGTTTTAGTGTAAAGCTCTAAATCTT
GCAAGATTATTCCCAGCATTAGGTACGACACATCTGGAAGAATCTGCCAACTGAGTGCTAGCAAGGAT
TACGCTGAGAAGGTAACAACTCAGTGCTTTCACTGTTAACCGTCCTCCACAACTGCTGTTTTTTGGTGT
TTTTGTGTGTGTGTGTTCGTTTGTTTCTTTGTTTTCCGAATAAATAAACAATACTTTGTTGAGCTC
